# Surrogate threshold effect based on a meta-analysis for the predictive value of progression-free survival for overall survival in hormone receptor-positive, HER2-negative metastatic breast cancer

**DOI:** 10.1007/s10549-019-05262-4

**Published:** 2019-05-07

**Authors:** Michael Patrick Lux, Sarah Böhme, Stephanie Hücherig, Ulli Jeratsch, Niclas Kürschner, Diana Lüftner

**Affiliations:** 1 Kooperatives Brustzentrum Paderborn, Paderborn, Germany; 2 Frauenklinik St. Louise, Paderborn, Germany; 3 St. Josefs-Krankenhaus, Salzkotten, Germany; 4 Frauen- und Kinderklinik St. Louise, Husener Str. 81, 33098 Paderborn, Germany; 5 Pfizer Deutschland GmbH, Linkstraße 10, 10785 Berlin, Germany; 6 AMS Advanced Medical Services GmbH, Rosa-Bavarese-Str. 5, 80639 Munich, Germany; 7 Klinik für Hämatologie, Onkologie und Tumorimmunologie, Charité – Universitätsmedizin Berlin, Campus Benjamin Franklin, Hindenburgdamm 30, 12200 Berlin, Germany

**Keywords:** Breast neoplasms, Surrogate threshold effect, Progression-free survival, Overall survival, Surrogate validation

## Abstract

**Purpose:**

Clinical trials investigating therapies for metastatic breast cancer (mBC) generally use progression-free survival (PFS) as primary endpoint, which is not accepted as patient-relevant outcome within the German benefit assessment. Hence a validation of PFS as surrogate endpoint for overall survival (OS) is needed, e.g., in the indication of HR+, HER2-negative mBC.

**Methods:**

A systematic search was conducted. RCT were included if at least one study arm investigated fulvestrant, letrozole, tamoxifen, exemestane, or anastrozole. Additionally, hazard ratios reported for OS/PFS including confidence interval or standard error were mandatory. Pearson correlation coefficient was calculated to estimate the relation of surrogate endpoint PFS and patient-relevant outcome OS as well as the surrogate threshold effect (STE) which is used to determine thresholds for the estimate of the surrogate endpoint.

**Results:**

16 studies with 5324 patients in total were included in the analyses. The correlation between hazard ratios of PFS and OS was statistically significant (*r* = 0.72, 95% CI 0.35–0.90) representing a positive linear relationship. STE analysis was applied. The meta-regression model revealed a STE for HR_PFS_ of 0.60 and sensitivity analyses underlined robustness of the results.

**Conclusions:**

Based on the derived STE, it is possible to draw conclusions on a significant effect in OS for a hypothetical trial demonstrating an upper confidence limit of HR_PFS_ < 0.60 in PFS. However, only final OS results are able to confirm if a clinical relevant difference in survival time can be achieved.

**Electronic supplementary material:**

The online version of this article (10.1007/s10549-019-05262-4) contains supplementary material, which is available to authorized users.

## Introduction

Endocrine therapies are the mainstay of treatment in hormone receptor-positive (HR+), human epidermal growth factor receptor 2 (HER2)-negative metastatic breast cancer (mBC) except in life-threatening situations qualifying the patient to receive chemotherapy [1].

Clinical trials investigating therapies for mBC often use progression-free survival (PFS) as primary endpoint [2], since patients with mBC have a relatively long survival time of around 3 years in median. With the desire to rapidly translate promising new agents into clinical practice, there is the need for endpoints which can be measured in a timely manner. Therefore, it is currently discussed whether endpoints based on disease progression, including PFS, time-to-progression (TTP), or time-to-treatment failure (TTF), are appropriate to demonstrate clinical benefit. These endpoints ensure an early availability of study outcomes and can serve as sensitive parameters for the benefit of a study medication as they are not influenced by subsequent lines of therapy or cross-over [2, 3]. Further advantages are the widespread use and comparability of PFS and TTP since they are most frequently used as primary endpoints in phase III trials and are worldwide accepted for the approval of new drugs [4–6].

However, the prolongation of overall survival (OS) is one of the most important therapeutic goals [7]. OS is regarded as unambiguous criterion, but there are certain disadvantages of OS as primary endpoint in the metastatic setting of breast cancer: the need for large numbers of patients, the long duration of follow-up phases until results become available, and the need for multiple subsequent therapies, which can confound OS. These limitations particularly cause difficulties in first-line studies [8, 9].

Health technology assessment (HTA) agencies worldwide generally accept PFS as endpoint in clinical trials [10], whereas the German Institute for Quality and Efficiency in Health Care (IQWiG) and the Federal Joint Committee (Gemeinsamer Bundesausschuss, G-BA) do not accept endpoints based on disease progression as a patient-relevant outcome within the benefit assessment of pharmaceuticals because they are measured by imaging techniques. Patient relevance of such endpoints might be accepted when measured via symptoms experienced by the patient. This would, however, lead to an omission of the re-evaluation of metastases in the course of clinical trials, which is considered unethical by physicians and does not comply with guideline recommendations [11]. Possible solutions for these different requirements have to be developed.

IQWiG suggested methods for the validation of surrogate endpoints in HTA context [12]. The aim of this study was the application of these methods in the indication of HR+, HER2-negative mBC to validate PFS as surrogate endpoint for OS.

## Materials and methods

### Literature search

A systematic search was conducted on the basis of the databases MEDLINE and EMBASE as well as in five EBM Reviews sources in September 2016 and was performed in accordance with PRISMA guidelines (Appendix A.1). The following keywords and associated subject headings were used: “breast cancer” and “metastatic” or “locally advanced” in combination with “fulvestrant” or “letrozole” or “tamoxifen” or “exemestane” or “anastrozole” (Online Appendices A.2–A.4). Inclusion criteria for trials are listed in Table 1.

**Table 1:** Inclusion criteria for trials in the systematic literature search

Population	Women with hormone receptor-positive and/or estrogen receptor-positive and/or progesterone receptor-positive, HER2-negative, locally advanced [not amenable to resection or radiotherapy with curative intent] or metastatic breast cancer regardless of line of treatment for locally advanced or metastatic disease
Intervention	At least one study arm investigated: fulvestrant, letrozole, tamoxifen, exemestane, or anastrozole
Comparator	Any drug intervention as single agent or in combination therapy
Endpoints	Overall survival and progression-free survival (according to RECIST)^a^ reported as hazard ratio of interventional study drug vs. control from a cox proportional hazard model and confidence interval or standard error
Type of study	Randomized controlled trials (all phases)
Publication type	Randomized controlled trials reported in accordance with CONSORT guidelines
Language	English, German

*CONSORT* Consolidated Standards of Reporting Trials, *HER2* human epidermal growth factor receptor 2, *RECIST* Response Evaluation Criteria In Solid Tumors, *TTP* time-to-progression

^a^TTP or comparable endpoints were considered if the definition was identical to PFS (time from randomization to objective disease progression or death from any cause)

Randomized controlled trials (RCT) were included if at least 80% of the study population met the inclusion criteria. In case of missing information regarding HER2 status or HR status, the proportion of patients meeting the inclusion criteria was extrapolated based on epidemiological data. In case HER2 status was unknown, a proportion of 81.9% of HR+ patients was assumed to be HER2-negative [13]; for patients with both unknown HER2 status and hormone receptor status, a proportion of 64.5% was assumed to be HR+ and HER2-negative [13]. Trials with TTP or comparable endpoints were considered if the definition was identical to PFS (time from randomization to objective disease progression or death from any cause). Only studies reporting PFS according to Response Evaluation Criteria In Solid Tumors (RECIST) [14] were included to ensure standardized and comparable endpoint evaluation. Overall survival had to be reported in the studies and should be defined as the time from the date of randomization to the date of death from any cause.

Two reviewers independently assessed citations to determine relevance to the research question. Included studies were cross-checked for relevance by physicians. If several publications for one study were available, data from the latest publication or publications reporting final data cuts were used. Data from included studies were extracted by one reviewer; another reviewer checked for consistency against the original source. Risk of bias on study level was assessed and summarized for all included individual studies (Online Appendix A.5).

### Statistical methods

As part of a rapid report, the German IQWiG presented methods for surrogate endpoints validation and recommendations for correlation-based procedures [12]. Health technology assessments are based on these methods in Germany. The methods include the evaluation of the certainty of conclusion of study results and the correlation between effect estimates of surrogate endpoint (e.g., PFS) and true outcome (e.g., OS) on trial level, whereas correlation is estimated by sample Pearson correlation coefficient r. Requirements for a successful surrogate validation are a high correlation (lower confidence limit (LCL) of *r* > 0.85) and a high certainty of conclusion of results of included studies. If the correlation is low (upper confidence limit < 0.7), no statement of surrogate validation is possible. In all other cases—where correlation is in the medium range and validity of surrogate endpoint is therefore unclear according to IQWiG methodology—they propose to apply the concept of STE [15], allowing conclusions on true endpoints by means of surrogate endpoints. STE is defined as minimal treatment effect on the surrogate endpoint explaining a non-zero (i.e., significant) effect on the true endpoint. In this context, STE represents the maximum value of the hazard ratio for PFS (HR_PFS_) that needs to be observed in a trial to ensure the possibility to draw conclusion of a significant effect on OS.

First, we tested the correlation between both outcomes (*H*_0_: *r* = 0 vs. *H*_1_: *r* ≠ 0). Second, if correlation was medium, we fitted a random effects mixed-model to the data with moderator HR_PFS_ and outcome variable hazard ratio of OS (HR_OS_) weighted by standard error (SE) of OS using the restricted maximum likelihood (REML) estimator for the amount of heterogeneity. Since SE is usually not reported, we recalculated it by means of 95% confidence interval (CI) of hazard ratio with (log(HR) − log(HR_LCL_))/*z*_(0.975)_, whereas *z*_(0.975)_ is the 97.5 percentile of the standard normal distribution. Based on the regression fit, we calculated a prediction band to a significance level of *α* = 0.05 for HR_OS_. Meta-regression model and prediction values were implemented with R [16] using functions rma.uni and predict.rma from metafor package [17]. The STE resulted from the intersection of the upper prediction limit curve and the horizontal where HR_OS_ = 1 (zero effect).

In sensitivity analyses, we investigated if factors HER2 status (reported vs. not reported), line of treatment (only first-line vs. others), and therapy option (studies comparing combination therapy with monotherapy vs. studies comparing two monotherapies) accounted for heterogeneity.

## Results

### Systematic literature search

**Fig. 1:**
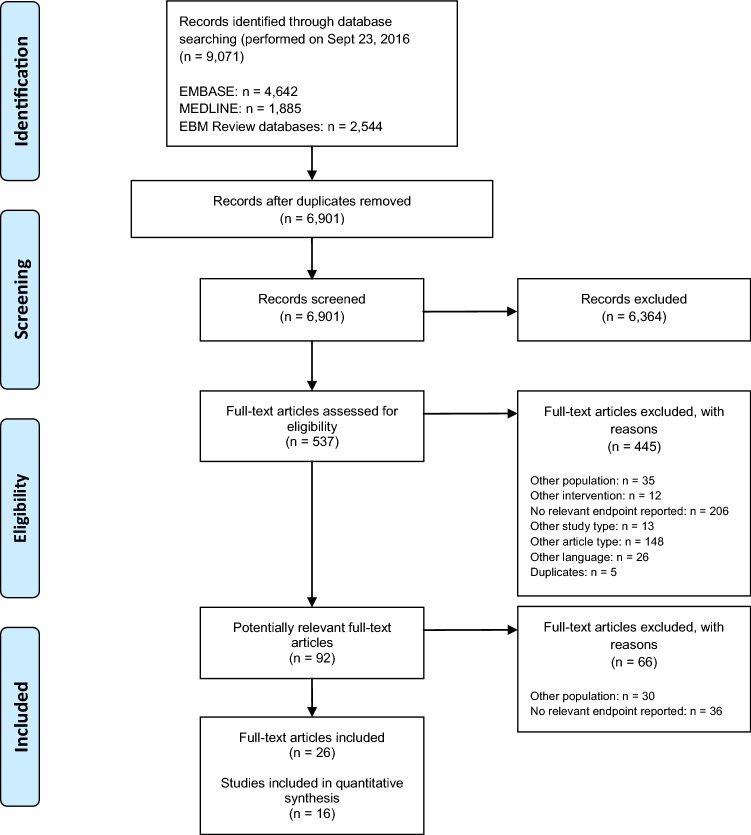
Flow diagram of study selection process. *N* Number of patients

The search identified 9071 citations from MEDLINE^®^, EMBASE, and EBM Review databases. We included 16 studies (26 full texts) for analysis (Fig. 1).

Characteristics for included trials are summarized in Table 2. The 16 trials included 5324 patients in total. In ten trials, HER2 status was reported for the entire study population. Six trials were included in the analysis since 80% of the study population met the inclusion criteria due to calculations according to epidemiological data (see methods). Six trials (2875 patients) evaluated treatments exclusively in the first-line setting for locally advanced or metastatic disease, and ten trials (2449 patients) included pretreated patients or patients in various lines of treatment. Almost all trials included postmenopausal women except for two trials which included a small (2.9%) [18] or unknown [19] number of premenopausal women treated with GnRH agonists.

**Table 2:** Overview of trial characteristics

No.	Primary Author, year of publication	Enrollment period	Patients with HER2-negative status (%)	Hormone receptor-positive status (%)	Double-blinded	Line of therapy^a^	Therapy option (c = combi vs. mono; m = mono vs. mono)	Intervention (study drug vs. control)	Median follow-up	HR OS (95% CI), *n*_A_ = number of events study drug, *n*_B_ = number of events control	HR PFS (95% CI), *n*_A_ = number of events study drug, *n*_B_ = number of events control
(1)	Bachelot, 2012 [20]^b^	March 2008–May 2009	95.5	100 (ER and/or PR)	No	1 + 2	c	Tamoxifen + everolimus (*n* = 54) vs. tamoxifen (*n* = 57)	Tamoxifen + everolimus: 23.7 months (range 2.6–32.7 months), tamoxifen: 24.2 months (range 0.9–36.2 months)	0.45 (0.24, 0.81), *n*_A_ = 16, *n*_B_ = 31	TTP, 0.54 (0.36, 0.81), *n*_A_ = NA, *n*_B_ = NA
(2)	Bergh, 2012 (FACT) [18]^b^	January 2004–March 2008	NA^c^	100 (ER and/or PR)	No	1	c	Anastrozole + fulvestrant (*n* = 258) vs. anastrozole (*n* = 256)	8.9 months (range 0–54 months)	1.00 (0.76, 1.32), *n*_A_ = 102, *n*_B_ = 102	TTP, 0.99 (0.81, 1.20), *n*_A_ = 200, *n*_B_ = 200
(3)	Burstein, 2014 (CALGB 40302) [21]^b^	September 2006–July 2010	80.8	100 (ER and/or PR)	Yes	1 + 2 + 3	c	Fulvestrant + lapatinib (*n* = 146) vs. fulvestrant + placebo (*n* = 145)	33.6 months	1.10 (0.83, 1.47)^d^, *n*_A_ = NA, *n*_B_ = NA	0.96 (0.75, 1.22)^d^, *n*_A_ = NA, *n*_B_ = NA
(4)	Clemons, 2014 (ZAMBONEY) [22]^b^	October 2009–October 2011	95.3	100 (ER and/or PR of primary tumor)	Yes	1 + 2 + 3	c	Fulvestrant + vandetanib (*n* = 61) vs. fulvestrant + Placebo (*n* = 68)	NA	0.69 (0.37, 1.31), *n*_A_ = 18, *n*_B_ = 23	0.94 (0.64, 1.36), *n*_A_ = 55, *n*_B_ = 63
(5)	Di Leo, 2010/2014 (CONFIRM)^e^ [23]^b^ [24]^b^	February 2005–August 2007	NA^c^	100 ER	Yes	1 + 2	m	Fulvestrant 500 mg (*n* = 362) vs. fulvestrant 250 mg (*n* = 374)	NA	0.79 (0.67, 0.94), *n*_A_ = 261, *n*_B_ = 293	0.79 (0.68, 0.93)^f^, *n*_A_ = 297, *n*_B_ = 321
(6)	Dickler, 2016 (CALGB 40503) [19]^b^	May 2008–November 2011	90.7	100 (ER and/or PR)	Yes	1 + 2	c	Letrozole + bevacizumab (*n* = 173) vs. letrozole + Placebo (*n* = 170)	39 months for PFS, 42 months for OS	0.87 (0.65, 1.18), *n*_A_ = 81, *n*_B_ = 90	0.75 (0.59, 0.96), *n*_A_ = 126, *n*_B_ = 138
(7)	Finn, 2015 (PALOMA-1) [25]^b^	December 2009–May 2012	100	100 (ER)	No	1	c	Letrozole + palbociclib (*n* = 84) vs. letrozole (*n* = 81)	Letrozole + palbociclib: 29.6 months (95% CI 27.9–36.0), letrozole: 27.9 months (95% CI 25.5–31.1)	0.81 (0.49, 1.34), *n*_A_ = 30, *n*_B_ = 31	0.49 (0.32, 0.75), *n*_A_ = 41, *n*_B_ = 59
(8)	Iwata, 2013 [26]^b^	April 2005–December 2010	~86.6^c^	88.66 (ER and/or PR)	Yes	1	m	Exemestane (*n* = 147) vs. anastrozole (*n* = 145)	NA	1.06 (0.73, 1.54), *n*_A_ = 57, *n*_B_ = 55	TTP, 1.01 (0.77, 1.32), *n*_A_ = 103, *n*_B_ = 114
(9)	Johnston, 2013 (SoFEA)^g^ [27]^b^	March 2004–August 2010	~86.6^h^	99.6% (ER and/or PR)	Yes (anastrozole and placebo)	1 + 2	c	Fulvestrant + anastrozole (*n* = 243) vs. fulvestrant + Placebo (*n* = 231)	37.9 months (interquartile range 23.1–50.8 months)	0.95 (0.76, 1.17), *n*_A_ = 168, *n*_B_ = 167	1.00 (0.83, 1.21), *n*_A_ = 235, *n*_B_ = 221
(10)	Llombart-Cussac, 2012 [28]^b^	September 2001–May 2003	NA^c^	100 (ER and/or PR)	No	1	m	Exemestane (*n* = 49) vs. Anastrozole (*n* = 51)	9.1 months (range, 0.07–79.96 months)	1.33 (0.78, 2.25), *n*_A_ = NA, *n*_B_ = NA	TTP, 1.13 (0.75, 1.72), *n*_A_ = 44, *n*_B_ = 44
(11)	Martin, 2015 (LEA) [29]^b^	November 2007–August 2011	100	100	No	1	c	Letrozole/fulvestrant + bevacizumab (*n* = 190) vs. letrozole/fulvestrant (*n* = 184)	23.7 months (range, 0–58.2 months)	0.87 (0.58, 1.32), *n*_A_ = 47, *n*_B_ = 45	0.83 (0.65, 1.06), *n*_A_ = 128, *n*_B_ = 135
(12)	Mehta, 2012^i^ [30]^b^	June 2004–July 2009	89.3^h^	100 (ER and/or PR)	No	1	c	Anastrozole + fulvestrant (*n* = 349) vs. anastrozole (*n* = 345)	35 months (range, 3–78) for PFS	0.81 (0.65, 1.00), *n*_A_ = 154, *n*_B_ = 176	0.80 (0.68, 0.94), *n*_A_ = 268, *n*_B_ = 297
(13)	Piccart, 2014 (BOLERO-2) [31]^b^	June 2009 – January 2011	100	100 (ER and/or PR)	Yes	1 + 2	c	Exemestane + everolimus (*n* = 485) vs. exemestane + placebo (*n* = 239)	NA	0.89 (0.73, 1.10), *n*_A_ = 267, *n*_B_ = 143	0.45 (0.38, 0.54), *n*_A_ = NA, *n*_B_ = NA
(14)	Robertson, 2013 [32]^b^	March 2008–July 2009	94.9	100 (ER and/or PR)	Yes (ganitumab/placebo)	1 + 2	c	Exemestane/fulvestrant + ganitumab (*n* = 106) vs. exemestane/fulvestrant + placebo (*n* = 50)	NA	1.78 (1.06, 2.98)^j^, *n*_A_ = 63, *n*_B_ = 19	1.17 (0.80, 1.72)^j^, *n*_A_ = NA, *n*_B_ = NA
(15)	Yamamoto, 2013 [33]^b^	October 2008–November 2011	91.2	100 (ER and/or PR)	No	≥ 2	m	Toremifene (*n* = 46) vs. exemestane (*n* = 45)	NA	0.60 (0.26, 1.39), *n*_A_ = NA, *n*_B_ = NA	0.61 (0.38, 0.99), *n*_A_ = NA, *n*_B_ = NA
(16)	Yardley, 2013 [34]^b^	June 2008–July 2010	90.8	98.5% (ER) 78.5% (PR)	Yes	≥ 1 NA	c	Exemestane + entinostat (*n* = 64) vs. exemestane + placebo (*n* = 66)	Exemestane + entinostat: 24.0 months, exemestane + placebo 26.4 months, for OS, respectively	0.59 (0.36, 0.97), *n*_A_ = NA, *n*_B_ = NA	0.73 (0.50, 1.07), *n*_A_ = NA, *n*_B_ = NA

*CI* confidence interval, *HR* hazard ratio of interventional study drug vs. control, *NA* not available

^a^Line of therapy for locally advanced or metastatic disease, previous therapy included endocrine and/or chemotherapy

^b^Publication reporting hazard ratio for relevant endpoints

^c^According to registry data, a proportion of 81.9% of the hormone receptor-positive patients was assumed to be HER2-negative in case HER2 status was unknown [13]. For patients with both unknown HER2 status and hormone receptor status, a proportion of 64.5% was assumed to be hormone receptor-positive and HER2-negative [13]

^d^Recalculated hazard ratio for lapatinib vs. placebo. Burstein, 2014 originally reported inverse effect measures of placebo to lapatinib: OS HR: 0.91; 95% CI: 0.68-1.21, PFS HR: 1.04; 95% CI: 0.82-1.33

^e^CONFIRM results for endpoint OS are published by Di Leo, 2010 and results for endpoint PFS are published by Di Leo, 2014

^f^Corrected HR and CI used for analyses [23]

^g^SoFEA also examined third treatment arm exemestane and compared fulvestrant + placebo vs. exemestane which is not included in STE analysis

^h^Study included both patients with known HER2 status as well as patients with unknown HER2 status (hormone receptor status of patients positive for both groups). For the latter group of patients, 81.9% was assumed to be HER2-negative; see footnote (d)

^i^Mehta, 2012 reported comparison of anastrozole vs. anastrozole + fulvestrant. To be consistent with other trials in the study pool comparing a combination therapy with a mono therapy, we calculated inverse hazard ratios to represent the comparison of anastrozole + fulvestrant vs. anastrozole

^j^Recalculated 95% CI to be consistent with other results of this table. Robertson, 2013 originally reported 80% CI for OS (HR 1.78, 80% CI 1.27–2.50; *p* = 0.025) and PFS (HR 1.17, 80% CI 0.91–1.50; *p* = 0.44)

Twelve trials compared combination therapy with monotherapy, while four trials compared monotherapy versus monotherapy. Combination treatments were add-on to hormone therapy and comprised different compound classes in comparison to endocrine therapy.

Endpoints were reported for intention-to-treat population (seven trials), full analysis set (three trials), modified intention-to-treat (two trials), or for all randomized patients (three trials). For one trial, no information was given on the analysis population.

### Statistical analysis

**Fig. 2:**
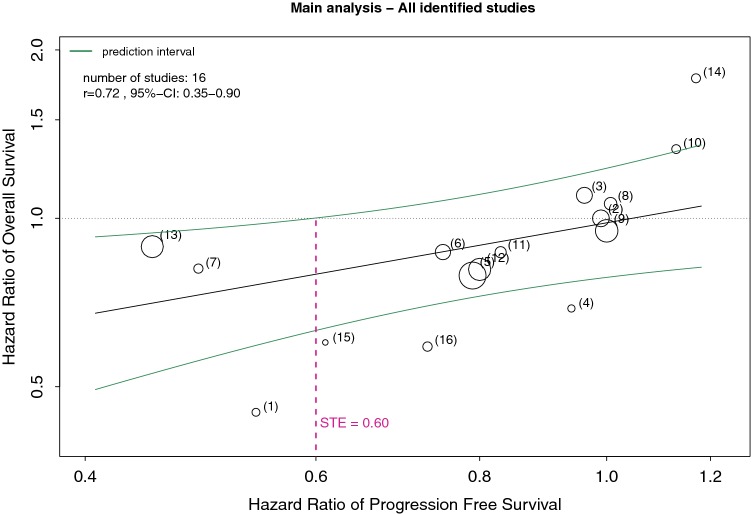
Meta-regression showing the relationship between hazard ratios of PFS and OS. Expansions of circles were scaled by the inverse of the standard error of HR_OS_. Numbers in parentheses reflect studies in Table 2. STE is defined as maximum value of HR_PFS_ so that HR_OS_ still is significant, i.e., upper confidence limit of HR_OS_ < 1. *CI* Confidence interval, *HR* hazard ration, *OS* overall survival, *PFS* progression-free survival, *r* Pearson correlation coefficient, *STE* surrogate threshold effect

In the main analysis (pool of 16 identified trials), the correlation between hazard ratios of PFS and OS was statistically significant (*r* = 0.72, 95% CI 0.35–0.90, *p* = 0.0016) representing a positive linear relationship of surrogate endpoint and by this patient-relevant endpoint. According to the definition in IQWiG’s rapid report, correlation was merely medium-sized and therefore the validity of the surrogate endpoint is unclear and a STE analysis is applied. The meta-regression showed low residual heterogeneity (*τ*^2^ = 0.009, *I*^2^ = 25%) and provided a significant result of the moderator coefficient *β*_PFS_ (*p* = 0.0206). STE for HR_PFS_ was 0.60 (Fig. 2), and thus for trials meeting the above-mentioned inclusion criteria in this specific indication and upper confidence limit of HR_PFS_ below STE, it is possible to draw the conclusion of a significant effect on OS by means of surrogate endpoint PFS.

Sensitivity analyses to check the robustness in the main analysis were performed to account for available information about HER2 status (sensitivity analysis 1), line of treatment (sensitivity analysis 2), or therapy option (sensitivity analysis 3) (Table 3). Due to the smaller sample sizes in the subpools, STE values deviate from the value in the main analysis, but correlation in all subpools is positive and at least of a medium magnitude, confirming the positive relationship between OS and PFS. In all subpools STE is below 1 except for sensitivity analysis 2b (Table 3). In this case, STE cannot be calculated (upper confidence limit of HR_OS_ > 1 for any value of HR_PFS_). Hence, meta-regression analyses in all specified subpools did not show heterogeneity regarding the observed factors and confirm the results of the main analysis.

**Table 3:** Overview of sensitivity analyses

Analysis	Pool	Number of studies	Correlation	STE
Main analysis	All identified studies	16	0.72	0.60
Sensitivity analysis 1	HER2 status			
	(a) Studies reporting HER2 status of patients	13	0.68	0.45
	(b) Studies not reporting HER2 status	3	n. c.^a^	0.86
Sensitivity analysis 2	Line of treatment			
	(a) Studies only including first-line patients	6	0.82	0.75
	(b) Studies including pretreated patients or patients in various lines	10	0.71	n.c.^b^
Sensitivity analysis 3	Therapy option			
	(a) Studies comparing a combination therapy with a mono therapy (combi vs. mono)	12	0.65	0.47
	(b) Studies comparing two monotherapies (mono vs. mono)	4	0.99	0.84

HER2 human epidermal growth factor receptor 2, *n.c.* not calculable

^a^In order to calculate a correlation coefficient, at least 4 studies are needed

^b^Upper confidence limit of HR_OS_ > 1 for any value of HR_PFS_

## Discussion

PFS is an accepted endpoint with a definition based on standardized criteria according to RECIST [14]. The outcome of PFS is not influenced by subsequent therapies, and results are timely available and a lower number of patients are needed than for OS. In addition, results are widely accepted for the approval [4, 5] as well as the HTA evaluation of new drugs [10] except from German HTA bodies due to an assumption of missing proof of patient relevance due to evaluation of PFS by imaging and not by symptoms.

From a physician’s point of view, PFS has a high relevance for patients. In case of a progression, the patient’s therapy needs to be changed, which entails different adverse effects and requires new procedures and adjustments of schedules. A proven progression also has a significant impact on the psychological well-being and quality of life [35].

Additionally, a prolongation of OS and maintaining quality of life continues to be the focus of treatment in the metastatic situation of breast cancer [7]. To quickly transfer results on PFS from trials with innovative therapies to clinical practice, it would be advantageous if a validation of progression-based endpoints as surrogate endpoint for OS is available, which was the aim of this study.

Methods used in this work have some limitations. It is possible that the pool of included studies does not include all publicly available data because the search was limited to three literature databases and included no further sources. In addition, several aspects often lead to exclusion of studies. One reason was poor reporting, for example if data for only one of the required endpoint were published. Lack of information regarding HER2 status leading to non-conformity with the defined patient population and no PFS/TTP assessment according to RECIST criteria were other reasons. Especially older studies were often not in accordance with the inclusion criteria.

The sensitivity analyses show that the STE values vary strongly when only very small study subsets are considered. Nevertheless, the values are not so far apart that they would point completely in the other direction, i.e., STE > 1. Furthermore, the STE is sensitive to outlier observations when number of studies in the model is low. The generation of randomization and whether allocation concealment was adequately carried out was rarely reported in the individual studies. To what extent this has an impact on the endpoints OS and PFS and finally on the STE remains unclear.

According to IQWiG’s method description, the entire 95% CI of PFS has to be below the STE in order to take into account the uncertainty with which both estimators are affected. Gillhaus et al. [36] described that this approach reduces the *α* error, but also considerably reduces the power of the STE concept. Statistical power could be increased using a lower *α* significance level (e.g., 0.1 or 0.2) for the prediction band of HR_OS_ in the meta-regression model. However, this assumption can only be made if the hypothetical trial is conducted in patients with HR+, HER2-negative mBC treated with endocrine therapies alone or in combination with other targeted treatments. The model does not intend to predict the outcome of OS concerning HR or differences in median of OS from the model.

In general, OS results always need a critical appraisal. Especially in mBC, an improvement of OS for a new therapy option is difficult to measure. Factors like the heterogeneity of the disease, therapy complexity with integration of local therapies (surgery, radiotherapy), and a wide range of systemic therapies as well as a long survival in the metastatic situation with numerous different sequential courses of therapy may have an impact on the results of OS. A model calculation has shown that the probability of demonstrating a significant OS benefit decreases to less than 30% for a post-progression survival (PPS) of more than 12 months [37]. However, survival of several years has been reached especially in mBC. In addition, depending on the required statistical power, thousands of patients need to be recruited to identify a survival benefit. In the age of individualized therapy with numerous specific subgroups, these studies are hardly feasible. The authors also conclude that the interpretation of OS is only useful, if the PPS is really short [37].

Additional points to take into account are the clinical relevance of OS results. The STE calculated in this publication only allows to draw conclusions on OS in the above-mentioned settings and about the statistical significance of OS. However, it is not possible to predict the differences in median survival times and its clinical relevance. Therefore, it is possible that the final result for OS is statistically significant in a trial but might not be considered clinically relevant. For example, a difference of 3 months in median OS is clinically relevant in an indication with very short survival times like metastatic pancreatic carcinoma [38]. MBC has comparably long survival times of 2–3 years [39] and a difference of 3 months in median OS would normally not be considered clinically relevant. Even if a meaningfully relevant difference in median OS was achieved, a proven prolongation of life with a simultaneous significant deterioration in the quality of life is not always a desirable therapeutic goal [40].

In conclusion, we were able to calculate the STE (0.60) allowing to draw conclusions on OS through the surrogate endpoint PFS besides minor methodological limitations in trials with HR+, HER2-negative mBC treated with endocrine therapies alone or on combination.

This means that for a hypothetical or future trial demonstrating upper confidence limit of HR_PFS_ < 0.60 in PFS it is possible to conclude on a significant effect in OS. However, only final OS results can confirm if a clinical relevant difference in survival time is reached. For future prospects, reflecting the current results in regard to ongoing clinical studies examining the addition of CDK 4/6 inhibitors to endocrine therapy will be desirable since they mostly lack of statistical significant, mature OS data for the time being. As long as OS results are not available, conclusions using STE may be drawn from PFS. To gain quick results on a new drug, PFS remains a relevant endpoint with high clinical relevance.

## Electronic supplementary material

Below is the link to the electronic supplementary material.

Supplementary material 1 (DOCX 61 kb)

Figure A.1 Sensitivity analysis 1a (EPS 49 kb)

Figure A.2 Sensitivity analysis 1b (EPS 26 kb)

Figure A.3 Sensitivity analysis 2a (EPS 45 kb)

Figure A.4 Sensitivity analysis 2b (EPS 49 kb)

Figure A.5 Sensitivity analysis 3a (EPS 49 kb)

Figure A.6 Sensitivity analysis 3b (EPS 36 kb)
