# Supplementary material for: Surrogate threshold effect based on a meta-analysis for the predictive value of progression-free survival for overall survival in hormone receptor-positive, HER2-negative metastatic breast cancer
Source: Breast Cancer Res Treat. 2019 May 7;176(3):495–506. doi: 10.1007/s10549-019-05262-4 (PMC6586722; doi:10.1007/s10549-019-05262-4)
Supplement: Supplementary file 1 — Supplementary material 1 (DOCX 61 kb) [file 10549_2019_5262_MOESM1_ESM.docx]

**Appendices**

Table A.1: PRISMA 2009 Checklist

| **Section/topic** | **#** | **Checklist item** | **Reported on page #** |
| --- | --- | --- | --- |
| **TITLE** | | |  |
| Title | 1 | Identify the report as a systematic review, meta-analysis, or both. | p1 |
| **ABSTRACT** | | |  |
| Structured summary | 2 | Provide a structured summary including, as applicable: background; objectives; data sources; study eligibility criteria, participants, and interventions; study appraisal and synthesis methods; results; limitations; conclusions and implications of key findings; systematic review registration number. | p3 |
| **INTRODUCTION** | | |  |
| Rationale | 3 | Describe the rationale for the review in the context of what is already known. | p5 |
| Objectives | 4 | Provide an explicit statement of questions being addressed with reference to participants, interventions, comparisons, outcomes, and study design (PICOS). | p5 |
| **METHODS** | | |  |
| Protocol and registration | 5 | Indicate if a review protocol exists, if and where it can be accessed (e.g., Web address), and, if available, provide registration information including registration number. | NA |
| Eligibility criteria | 6 | Specify study characteristics (e.g., PICOS, length of follow-up) and report characteristics (e.g., years considered, language, publication status) used as criteria for eligibility, giving rationale. | pp6-7 |
| Information sources | 7 | Describe all information sources (e.g., databases with dates of coverage, contact with study authors to identify additional studies) in the search and date last searched. | pp6-7 |
| Search | 8 | Present full electronic search strategy for at least one database, including any limits used, such that it could be repeated. | Table A.2-A.4 |
| Study selection | 9 | State the process for selecting studies (i.e., screening, eligibility, included in systematic review, and, if applicable, included in the meta-analysis). | pp6-7 |
| Data collection process | 10 | Describe method of data extraction from reports (e.g., piloted forms, independently, in duplicate) and any processes for obtaining and confirming data from investigators. | p7 |
| Data items | 11 | List and define all variables for which data were sought (e.g., PICOS, funding sources) and any assumptions and simplifications made. | pp6-7 |
| Risk of bias in individual studies | 12 | Describe methods used for assessing risk of bias of individual studies (including specification of whether this was done at the study or outcome level), and how this information is to be used in any data synthesis. | pp6-7 |
| Summary measures | 13 | State the principal summary measures (e.g., risk ratio, difference in means). | pp6-7 |
| Synthesis of results | 14 | Describe the methods of handling data and combining results of studies, if done, including measures of consistency (e.g., I^2^) for each meta-analysis. | p7 |

| **Section/topic** | **#** | **Checklist item** | **Reported on page #** |
| --- | --- | --- | --- |
| Risk of bias across studies | 15 | Specify any assessment of risk of bias that may affect the cumulative evidence (e.g., publication bias, selective reporting within studies). | p7 |
| Additional analyses | 16 | Describe methods of additional analyses (e.g., sensitivity or subgroup analyses, meta-regression), if done, indicating which were pre-specified. | p7 |
| **RESULTS** | | |  |
| Study selection | 17 | Give numbers of studies screened, assessed for eligibility, and included in the review, with reasons for exclusions at each stage, ideally with a flow diagram. | p7  Figure 1  Table A.6  Table A.7 |
| Study characteristics | 18 | For each study, present characteristics for which data were extracted (e.g., study size, PICOS, follow-up period) and provide the citations. | p9  Table 2 |
| Risk of bias within studies | 19 | Present data on risk of bias of each study and, if available, any outcome level assessment (see item 12). | Table A.5 |
| Results of individual studies | 20 | For all outcomes considered (benefits or harms), present, for each study: (a) simple summary data for each intervention group (b) effect estimates and confidence intervals, ideally with a forest plot. | pp9  Table 2 |
| Synthesis of results | 21 | Present results of each meta-analysis done, including confidence intervals and measures of consistency. | p12  Table 3  Figure A.1-6 |
| Risk of bias across studies | 22 | Present results of any assessment of risk of bias across studies (see Item 15). | p12 |
| Additional analysis | 23 | Give results of additional analyses, if done (e.g., sensitivity or subgroup analyses, meta-regression [see Item 16]). | p12  Table 3  Figure A.1‑6 |
| **DISCUSSION** | | |  |
| Summary of evidence | 24 | Summarize the main findings including the strength of evidence for each main outcome; consider their relevance to key groups (e.g., healthcare providers, users, and policy makers). | p14 |
| Limitations | 25 | Discuss limitations at study and outcome level (e.g., risk of bias), and at review-level (e.g., incomplete retrieval of identified research, reporting bias). | p14 |
| Conclusions | 26 | Provide a general interpretation of the results in the context of other evidence, and implications for future research. | pp14-15 |
| **FUNDING** | | |  |
| Funding | 27 | Describe sources of funding for the systematic review and other support (e.g., supply of data); role of funders for the systematic review. | p16 |

*From:*  Moher D, Liberati A, Tetzlaff J, Altman DG, The PRISMA Group (2009). Preferred Reporting Items for Systematic Reviews and Meta-Analyses: The PRISMA Statement. PLoS Med 6(7): e1000097. doi:10.1371/journal.pmed1000097

For more information, visit: **www.prisma-statement.org**.

Table A.2: Search strategy Embase.^1^

| # | Searches | Results |
| --- | --- | --- |
| 1 | exp breast cancer/ or exp breast tumor/ or exp Breast Neoplasms/ or exp breast carcinoma/ or exp breast carcinogenesis/ | 434013 |
| 2 | (breast cancer* or breast car#inoma* or breast neoplas*).mp. | 428977 |
| 3 | ((breast* or mamma*) and (cancer* or car#ino* or adenocar#ino* or sarcom* or malignan* or tumo?r* or neoplasm*)).mp. | 576761 |
| 4 | (breast* or mamma*).mp. and exp neoplasm/ | 510993 |
| 5 | 1 or 2 or 3 or 4 | 585005 |
| 6 | exp metastasis/ or exp advanced cancer/ or exp recurrent disease/ | 691518 |
| 7 | (advanc* or metasta* or recur* or progress* or secondar* or relaps* or inoperab* or refractor* or incur* or disseminat*).mp. | 4033982 |
| 8 | ((late adj1 stage*) or (end adj1 stage*) or (stage adj1 "3*") or (stage adj1 "4*") or (stage adj1 IV*) or (stage adj1 III*)).mp. | 210560 |
| 9 | (abc or a-bc or mbc or m-bc).mp. | 46237 |
| 10 | 6 or 7 or 8 or 9 | 4180642 |
| 11 | exp fulvestrant/ or (fulvestrant* or faslodex* or ZD9238* or ZD 9238* or ICI 182* or ici 182780* or ici182780* or zd 182780* or zd182780* or zm182780* or zm182780*).mp. or 129453-61-8.rn. | 7314 |
| 12 | exp letrozole/ or (letrozol* or CGS 20267* or CGS20267* or FEM345* or FEM 345* or femar*).mp. or 112809-51-5.rn. | 9104 |
| 13 | exp tamoxifen/ or (tamoxifen* or nsc 180973* or nsc180973* or ici46474* or ici 46474* or ici47699* or ici 47699* or kessar* or tamoplac* or tamoxasta* or nolvadex* or Istubal* or Valodex* or tomaxithen* or zitazonium* or soltamox*).mp. or (10540-29-1 or 54965-24-1).rn. | 56307 |
| 14 | exp anastrozole/ or (anastrozol* or ZD 1033* or ZD1033* or ICI D1033* or ICID1033* or arimidex* or trozolet* or asiolex*).mp. or 120511-73-1.rn. | 8033 |
| 15 | exp exemestane/ or (exemestan* or fce 24304* or fce24304* or PNU 155971* or PNU155971* or aromasin* or nikidess*).mp. or 107868-30-4.rn. | 5010 |
| 16 | 11 or 12 or 13 or 14 or 15 | 66575 |
| 17 | random*.tw. or placebo*.mp. or double-blind*.tw. | 1359656 |
| 18 | 5 and 10 and 16 and 17 | 4592 |
| 1: Embase Classic+Embase | | |

Table A.3: Search strategy Medline.^1^

| # | Searches | Results |
| --- | --- | --- |
| 1 | exp Breast Neoplasms/ or exp Carcinoma, Intraductal, Noninfiltrating/ or exp Inflammatory Breast Neoplasms/ | 247576 |
| 2 | (breast cancer* or breast car#inoma* or breast neoplas*).mp. | 307063 |
| 3 | ((breast* or mamma*) and (cancer* or car#ino* or adenocar#ino* or sarcom* or malignan* or tumo?r* or neoplasm*)).mp. | 403665 |
| 4 | (breast* or mamma*).mp. and exp neoplasms/ | 323651 |
| 5 | 1 or 2 or 3 or 4 | 408101 |
| 6 | exp Neoplasm Metastasis/ or exp recurrence/ | 333276 |
| 7 | (advanc* or metasta* or recur* or progress* or secondar* or relaps* or inoperab* or refractor* or incur* or disseminat*).mp. | 2964735 |
| 8 | ((late adj1 stage*) or (end adj1 stage*) or (stage adj1 "3*") or (stage adj1 "4*") or (stage adj1 IV*) or (stage adj1 III*)).mp. | 137333 |
| 9 | (abc or a-bc or mbc or m-bc).mp. | 26137 |
| 10 | 6 or 7 or 8 or 9 | 3064433 |
| 11 | (fulvestrant* or faslodex* or ZD9238* or ZD 9238* or ICI 182* or ici 182780* or ici182780* or zd 182780* or zd182780* or zm 182780* or zm182780*).mp. or 129453-61-8.rn. | 3856 |
| 12 | (letrozol* or CGS 20267* or CGS20267* or FEM345* or FEM 345* or femar*).mp. or 112809-51-5.rn. | 2527 |
| 13 | exp tamoxifen/ or (tamoxifen* or nsc 180973* or nsc180973* or ici46474* or ici 46474* or ici47699* or ici 47699* or kessar* or tamoplac* or tamoxasta* or nolvadex* or Istubal* or Valodex* or tomaxithen* or zitazonium* or soltamox*).mp. or (10540-29-1 or 54965-24-1).rn. | 26441 |
| 14 | (anastrozol* or ZD 1033* or ZD1033* or ICI D1033* or ICID1033* or arimidex* or trozolet* or asiolex*).mp. or 120511-73-1.rn. | 1916 |
| 15 | (exemestan* or fce 24304* or fce24304* or PNU 155971* or PNU155971* or aromasin* or nikidess*).mp. or 107868-30-4.rn. | 1183 |
| 16 | 11 or 12 or 13 or 14 or 15 | 31542 |
| 17 | randomized controlled trial.pt. or randomized.mp. or placebo.mp. | 727171 |
| 18 | 5 and 10 and 16 and 17 | 1876 |
| 1: Epub Ahead of Print, In-Process & Other Non-Indexed Citations, Ovid MEDLINE(R) Daily and Ovid MEDLINE(R) 1946 to Present | | |

Table A.4: Search strategy EBM Reviews Databases.^1^

| # | Searches | Results |
| --- | --- | --- |
| 1 | exp Breast Neoplasms/ or exp Carcinoma, Intraductal, Noninfiltrating/ or exp Inflammatory Breast Neoplasms/ | 8845 |
| 2 | (breast cancer* or breast car#inoma* or breast neoplas*).mp. | 21072 |
| 3 | ((breast* or mamma*) and (cancer* or car#ino* or adenocar#ino* or sarcom* or malignan* or tumo?r* or neoplasm*)).mp. | 22559 |
| 4 | (breast* or mamma*).mp. and exp neoplasms/ | 9547 |
| 5 | 1 or 2 or 3 or 4 | 22578 |
| 6 | exp Neoplasm Metastasis/ or exp recurrence/ | 13910 |
| 7 | (advanc* or metasta* or recur* or progress* or secondar* or relaps* or inoperab* or refractor* or incur* or disseminat*).mp. | 178681 |
| 8 | ((late adj1 stage*) or (end adj1 stage*) or (stage adj1 "3*") or (stage adj1 "4*") or (stage adj1 IV*) or (stage adj1 III*)).mp. | 10828 |
| 9 | (abc or a-bc or mbc or m-bc).mp. | 2800 |
| 10 | 6 or 7 or 8 or 9 | 184243 |
| 11 | (fulvestrant* or faslodex* or ZD9238* or ZD 9238* or ICI 182* or ici 182780* or ici182780* or zd 182780* or zd182780* or zm 182780* or zm182780*).mp. or 129453-61-8.rn. | 215 |
| 12 | (letrozol* or CGS 20267* or CGS20267* or FEM345* or FEM 345* or femar*).mp. or 112809-51-5.rn. | 898 |
| 13 | exp tamoxifen/ or (tamoxifen* or nsc 180973* or nsc180973* or ici46474* or ici 46474* or ici47699* or ici 47699* or kessar* or tamoplac* or tamoxasta* or nolvadex* or Istubal* or Valodex* or tomaxithen* or zitazonium* or soltamox*).mp. or (10540-29-1 or 54965-24-1).rn. | 3928 |
| 14 | (anastrozol* or ZD 1033* or ZD1033* or ICI D1033* or ICID1033* or arimidex* or trozolet* or asiolex*).mp. or 120511-73-1.rn. | 756 |
| 15 | (exemestan* or fce 24304* or fce24304* or PNU 155971* or PNU155971* or aromasin* or nikidess*).mp. or 107868-30-4.rn. | 488 |
| 16 | 11 or 12 or 13 or 14 or 15 | 4975 |
| 17 | 5 and 10 and 16 | 2515 |
| 1: EBM Reviews - Cochrane Central Register of Controlled Trials June 2016, EBM Reviews - Cochrane Database of Systematic Reviews 2005 to July 27, 2016, EBM Reviews - Database of Abstracts of Reviews of Effects 1st Quarter 2016, EBM Reviews - Health Technology Assessment 2nd Quarter 2016, EBM Reviews - NHS Economic Evaluation Database 1st Quarter 2016 | | |

Table A.5: Risk of bias assessment on study level for all included studies

|  | Study | Random sequence generation | Allocation  concealment | Blinding | | | | No clues to result-controlled reporting | | No other aspects that can cause bias | | Bias potential at  study level | |
| --- | --- | --- | --- | --- | --- | --- | --- | --- | --- | --- | --- | --- | --- |
| # |  |  |  | Participant | | Therapist | |  |  |  |  |  |  |
|  | Bachelot et al., 2012 | unclear | unclear | | no | | no | | yes | | yes | | low |
|  | Bergh et al., 2012 (FACT) | yes | yes | | no | | no | | yes | | yes | | low |
|  | Burstein et al., 2014 (CALGB 40302) | unclear | unclear | | yes | | yes | | yes | | yes | | low |
|  | Clemons et al., 2014 (ZAMBONEY) | yes | yes | | yes | | yes | | yes | | yes | | low |
|  | Di Leo et al., 2010/2014 (CONFIRM) | yes | yes | | yes | | yes | | yes | | yes | | low |
|  | Dickler et al., 2016 (CALGB 40503) | yes | unclear | | yes^a^ | | yes^a^ | | yes | | yes | | low |
|  | Finn et al., 2015  (PALOMA-1) | yes | yes | | no | | no | | yes | | yes | | low |
|  | Iwata et al., 2013 | unclear | unclear | | yes | | yes | | yes | | yes | | low |
|  | Johnston et al., 2013 (SoFEA) | unclear | unclear | | yes | | yes | | yes | | yes | | low |
|  | Llombart-Cussac et al., 2012 | yes | unclear | | no | | no | | yes | | yes | | low |
|  | Martin et al., 2015 (LEA) | unclear | yes | | no | | no | | yes | | yes | | low |
|  | Mehta et al., 2012 | yes | yes | | no | | no | | yes | | yes | | low |
|  | Piccart et al., 2014 (BOLERO-2) | yes | unclear | | yes | | yes | | yes | | yes | | low |
|  | Robertson et al., 2013 | yes | yes | | yes | | yes | | yes | | yes | | low |
|  | Yamamoto et al., 2013 | unclear | unclear | | no | | no | | yes | | yes | | low |
|  | Yardley et al., 2013 | yes | yes | | yes | | yes | | yes | | yes | | low |
| a: Study started initially as double-blind, protocol amendment lead to open-lapel design | | | | | | | | | | | | | |

Table A.6: Pool of included trials and respective references.

| # | **Study**  References |
| --- | --- |
|  | **Bachelot, 2012**  Bachelot T, Bourgier C, Cropet C, Ray-Coquard I, Ferrero JM, Freyer G, et al. Randomized phase II trial of everolimus in combination with tamoxifen in patients with hormone receptor-positive, human epidermal growth factor receptor 2-negative metastatic breast cancer with prior exposure to aromatase inhibitors: a GINECO study. J Clin Oncol. 2012;30(22):2718-24. Epub 2012/05/09. |
|  | **Bergh, 2012 (FACT)**  Bergh J, Jonsson PE, Lidbrink EK, Trudeau M, Eiermann W, Brattstrom D, et al. FACT: an open-label randomized phase III study of fulvestrant and anastrozole in combination compared with anastrozole alone as first-line therapy for patients with receptor-positive postmenopausal breast cancer. J Clin Oncol. 2012;30(16):1919-25. Epub 2012/03/01. |
|  | **Burstein, 2014 (CALGB 40302)**  Burstein HJ, Cirrincione CT, Barry WT, Chew HK, Tolaney SM, Lake DE, et al. Endocrine therapy with or without inhibition of epidermal growth factor receptor and human epidermal growth factor receptor 2: a randomized, double-blind, placebo-controlled phase III trial of fulvestrant with or without lapatinib for postmenopausal women with hormone receptor-positive advanced breast cancer-CALGB 40302 (Alliance). J Clin Oncol. 2014;32(35):3959-66. Epub 2014/10/29. |
|  | **Clemons, 2014 (ZAMBONEY)**  Clemons MJ, Cochrane B, Pond GR, Califaretti N, Chia SK, Dent RA, et al. Randomised, phase II, placebo-controlled, trial of fulvestrant plus vandetanib in postmenopausal women with bone only or bone predominant, hormone-receptor-positive metastatic breast cancer (MBC): the OCOG ZAMBONEY study. Breast Cancer Res Treat. 2014;146(1):153-62. Epub 2014/06/14.  Addison CL, Pond GR, Cochrane B, Zhao H, Chia SK, Levine MN, et al. Correlation of baseline biomarkers with clinical outcomes and response to fulvestrant with vandetanib or placebo in patients with bone predominant metastatic breast cancer: An OCOG ZAMBONEY sub-study. J Bone Oncol. 2015;4(2):47-53. Epub 2015/11/19. |
|  | **Di Leo, 2010/2014 (CONFIRM)**  Di Leo A, Jerusalem G, Petruzelka L, Torres R, Bondarenko IN, Khasanov R, et al. Results of the CONFIRM phase III trial comparing fulvestrant 250 mg with fulvestrant 500 mg in postmenopausal women with estrogen receptor-positive advanced breast cancer. J Clin Oncol. 2010;28(30):4594-600. Epub 2010/09/22.  Di Leo A, Jerusalem G, Petruzelka L, Torres R, Bondarenko IN, Khasanov R, et al. Final overall survival: fulvestrant 500 mg vs 250 mg in the randomized CONFIRM trial. J Natl Cancer Inst. 2014;106(1):djt337. Epub 2013/12/10. |
|  | **Dickler, 2016 (CALGB 40503)**  Dickler MN, Barry WT, Cirrincione CT, Ellis MJ, Moynahan ME, Innocenti F, et al. Phase III Trial Evaluating Letrozole As First-Line Endocrine Therapy With or Without Bevacizumab for the Treatment of Postmenopausal Women With Hormone Receptor-Positive Advanced-Stage Breast Cancer: CALGB 40503 (Alliance). J Clin Oncol. 2016;34(22):2602-9. Epub 2016/05/04. |
|  | **Finn, 2015 (PALOMA-1)**  Finn RS, Crown JP, Ettl J, Schmidt M, Bondarenko IM, Lang I, et al. Efficacy and safety of palbociclib in combination with letrozole as first-line treatment of ER-positive, HER2-negative, advanced breast cancer: expanded analyses of subgroups from the randomized pivotal trial PALOMA-1/TRIO-18. Breast Cancer Res. 2016;18(1):67. Epub 2016/06/29.  Finn RS, Crown JP, Lang I, Boer K, Bondarenko IM, Kulyk SO, et al. The cyclin-dependent kinase 4/6 inhibitor palbociclib in combination with letrozole versus letrozole alone as first-line treatment of oestrogen receptor-positive, HER2-negative, advanced breast cancer (PALOMA-1/TRIO-18): a randomised phase 2 study. Lancet Oncol. 2015;16(1):25-35. Epub 2014/12/20. |
|  | **Iwata, 2013**  Iwata H, Masuda N, Ohno S, Rai Y, Sato Y, Ohsumi S, et al. A randomized, double-blind, controlled study of exemestane versus anastrozole for the first-line treatment of postmenopausal Japanese women with hormone-receptor-positive advanced breast cancer. Breast Cancer Res Treat. 2013;139(2):441-51. Epub 2013/05/30. |
|  | **Johnston, 2013 (SoFEA)**  Johnston SR, Kilburn LS, Ellis P, Dodwell D, Cameron D, Hayward L, et al. Fulvestrant plus anastrozole or placebo versus exemestane alone after progression on non-steroidal aromatase inhibitors in postmenopausal patients with hormone-receptor-positive locally advanced or metastatic breast cancer (SoFEA): a composite, multicentre, phase 3 randomised trial. Lancet Oncol. 2013;14(10):989-98. Epub 2013/08/02. |
|  | **Llombart-Cussac, 2012**  Llombart-Cussac A, Ruiz A, Anton A, Barnadas A, Antolin S, Ales-Martinez JE, et al. Exemestane versus anastrozole as front-line endocrine therapy in postmenopausal patients with hormone receptor-positive, advanced breast cancer: final results from the Spanish Breast Cancer Group 2001-03 phase 2 randomized trial. Cancer. 2012;118(1):241-7. Epub 2011/07/01. |
|  | **Martin, 2015 (LEA)**  Martin M, Loibl S, von Minckwitz G, Morales S, Martinez N, Guerrero A, et al. Phase III trial evaluating the addition of bevacizumab to endocrine therapy as first-line treatment for advanced breast cancer: the letrozole/fulvestrant and avastin (LEA) study. J Clin Oncol. 2015;33(9):1045-52. Epub 2015/02/19. |
|  | **Mehta, 2012**  Mehta RS, Barlow WE, Albain KS, Vandenberg TA, Dakhil SR, Tirumali NR, et al. Combination anastrozole and fulvestrant in metastatic breast cancer. N Engl J Med. 2012;367(5):435-44. Epub 2012/08/03. |
|  | **Piccart, 2014 (BOLERO-2)**  Piccart M, Hortobagyi GN, Campone M, Pritchard KI, Lebrun F, Ito Y, et al. Everolimus plus exemestane for hormone-receptor-positive, human epidermal growth factor receptor-2-negative advanced breast cancer: overall survival results from BOLERO-2dagger. Ann Oncol. 2014;25(12):2357-62. Epub 2014/09/19.  Baselga J, Campone M, Piccart M, Burris HA, 3rd, Rugo HS, Sahmoud T, et al. Everolimus in postmenopausal hormone-receptor-positive advanced breast cancer. N Engl J Med. 2012;366(6):520-9. Epub 2011/12/14.  Beaver JA, Park BH. The BOLERO-2 trial: the addition of everolimus to exemestane in the treatment of postmenopausal hormone receptor-positive advanced breast cancer. Future Oncol. 2012;8(6):651-7. Epub 2012/07/07.  Campone M, Bachelot T, Gnant M, Deleu I, Rugo HS, Pistilli B, et al. Effect of visceral metastases on the efficacy and safety of everolimus in postmenopausal women with advanced breast cancer: subgroup analysis from the BOLERO-2 study. Eur J Cancer. 2013;49(12):2621-32. Epub 2013/06/06.  Noguchi S, Masuda N, Iwata H, Mukai H, Horiguchi J, Puttawibul P, et al. Efficacy of everolimus with exemestane versus exemestane alone in Asian patients with HER2-negative, hormone-receptor-positive breast cancer in BOLERO-2. Breast Cancer. 2014;21(6):703-14. Epub 2013/02/14.  Pritchard KI, Burris HA, 3rd, Ito Y, Rugo HS, Dakhil S, Hortobagyi GN, et al. Safety and efficacy of everolimus with exemestane vs. exemestane alone in elderly patients with HER2-negative, hormone receptor-positive breast cancer in BOLERO-2. Clin Breast Cancer. 2013;13(6):421-32 e8. Epub 2013/11/26.  Yardley DA, Noguchi S, Pritchard KI, Burris HA, 3rd, Baselga J, Gnant M, et al. Everolimus plus exemestane in postmenopausal patients with HR(+) breast cancer: BOLERO-2 final progression-free survival analysis. Adv Ther. 2013;30(10):870-84. Epub 2013/10/26.  Chandarlapaty S, Chen D, He W, Sung P, Samoila A, You D, et al. Prevalence of ESR1 Mutations in Cell-Free DNA and Outcomes in Metastatic Breast Cancer: A Secondary Analysis of the BOLERO-2 Clinical Trial. JAMA Oncol. 2016. Epub 2016/08/18. |
|  | **Robertson, 2013**  Robertson JF, Ferrero JM, Bourgeois H, Kennecke H, de Boer RH, Jacot W, et al. Ganitumab with either exemestane or fulvestrant for postmenopausal women with advanced, hormone-receptor-positive breast cancer: a randomised, controlled, double-blind, phase 2 trial. Lancet Oncol. 2013;14(3):228-35. Epub 2013/02/19. |
|  | **Yamamoto, 2013**  Yamamoto Y, Ishikawa T, Hozumi Y, Ikeda M, Iwata H, Yamashita H, et al. Randomized controlled trial of toremifene 120 mg compared with exemestane 25 mg after prior treatment with a non-steroidal aromatase inhibitor in postmenopausal women with hormone receptor-positive metastatic breast cancer. BMC Cancer. 2013;13:239. Epub 2013/05/18. |
|  | **Yardley, 2013**  Yardley DA, Ismail-Khan RR, Melichar B, Lichinitser M, Munster PN, Klein PM, et al. Randomized phase II, double-blind, placebo-controlled study of exemestane with or without entinostat in postmenopausal women with locally recurrent or metastatic estrogen receptor-positive breast cancer progressing on treatment with a nonsteroidal aromatase inhibitor. J Clin Oncol. 2013;31(17):2128-35. Epub 2013/05/08. |

Table A.7: Fulltext screening of potential relevant references for inclusion in STE analyses or reason for exclusion

| Studie | Study included / excluded | Reason for exclusion | Reference(s) | Data from publication used (yes/no) |
| --- | --- | --- | --- | --- |
| Bachelot 2012 | included | - | Bachelot T., Bourgier C., Cropet C., Ray-Coquard I., Ferrero J. M., Freyer G., Abadie-Lacourtoisie S., Eymard J. C., Debled M., Spaeth D., Legouffe E., Allouache D., El Kouri C. and Pujade-Lauraine E.. 2012. Randomized phase II trial of everolimus in combination with tamoxifen in patients with hormone receptor-positive, human epidermal growth factor receptor 2-negative metastatic breast cancer with prior exposure to aromatase inhibitors: A GINECO study. Journal of Clinical Oncology, 30(22): 2718-2724 | yes |
|  | | | | |
| BOLERO-2 | included | - | Baselga J., Campone M., Piccart M., Burris H. A., 3rd, Rugo H. S., Sahmoud T., Noguchi S., Gnant M., Pritchard K. I., Lebrun F., Beck J. T., Ito Y., Yardley D., Deleu I., Perez A., Bachelot T., Vittori L., Xu Z., Mukhopadhyay P., Lebwohl D. and Hortobagyi G. N.. 2012. Everolimus in postmenopausal hormone-receptor-positive advanced breast cancer. New England Journal of Medicine, 366(6): 520-9 | no |
|  |  |  | Beaver J. A. and Park B. H.. 2012. The BOLERO-2 trial: the addition of everolimus to exemestane in the treatment of postmenopausal hormone receptor-positive advanced breast cancer. Future Oncology, 8(6): 651-7 | no |
|  |  |  | Campone M., Bachelot T., Gnant M., Deleu I., Rugo H. S., Pistilli B., Noguchi S., Shtivelband M., Pritchard K. I., Provencher L., Burris H. A., 3rd, Hart L., Melichar B., Hortobagyi G. N., Arena F., Baselga J., Panneerselvam A., Heniquez A., El-Hashimyt M., Taran T., Sahmoud T. and Piccart M.. 2013. Effect of visceral metastases on the efficacy and safety of everolimus in postmenopausal women with advanced breast cancer: subgroup analysis from the BOLERO-2 study. European Journal of Cancer, 49(12): 2621-32 | no |
|  |  |  | Noguchi S., Masuda N., Iwata H., Mukai H., Horiguchi J., Puttawibul P., Srimuninnimit V., Tokuda Y., Kuroi K., Iwase H., Inaji H., Ohsumi S., Noh W. C., Nakayama T., Ohno S., Rai Y., Park B. W., Panneerselvam A., El-Hashimy M., Taran T., Sahmoud T. and Ito Y.. 2014. Efficacy of everolimus with exemestane versus exemestane alone in Asian patients with HER2-negative, hormone-receptor-positive breast cancer in BOLERO-2. Breast cancer (Tokyo, Japan), 21(6): 703-714 | no |
|  |  |  | Piccart M., Hortobagyi G. N., Campone M., Pritchard K. I., Lebrun F., Ito Y., Noguchi S., Perez A., Rugo H. S., Deleu I., Burris H. A., 3rd, Provencher L., Neven P., Gnant M., Shtivelband M., Wu C., Fan J., Feng W., Taran T. and Baselga J.. 2014. Everolimus plus exemestane for hormone-receptor-positive, human epidermal growth factor receptor-2-negative advanced breast cancer: overall survival results from BOLERO-2+. Annals of Oncology, 25(12): 2357-62 | yes |
|  |  |  | Pritchard K. I., Burris H. A., 3rd, Ito Y., Rugo H. S., Dakhil S., Hortobagyi G. N., Campone M., Csoszi T., Baselga J., Puttawibul P., Piccart M., Heng D., Noguchi S., Srimuninnimit V., Bourgeois H., Gonzalez Martin A., Osborne K., Panneerselvam A., Taran T., Sahmoud T. and Gnant M.. 2013. Safety and efficacy of everolimus with exemestane vs. exemestane alone in elderly patients with HER2-negative, hormone receptor-positive breast cancer in BOLERO-2. Clinical Breast Cancer, 13(6): 421-432.e8 | no |
|  |  |  | Yardley D. A., Noguchi S., Pritchard K. I., Burris H. A., 3rd, Baselga J., Gnant M., Hortobagyi G. N., Campone M., Pistilli B., Piccart M., Melichar B., Petrakova K., Arena F. P., Erdkamp F., Harb W. A., Feng W., Cahana A., Taran T., Lebwohl D. and Rugo H. S.. 2013. Everolimus plus exemestane in postmenopausal patients with HR(+) breast cancer: BOLERO-2 final progression-free survival analysis.[Erratum appears in Adv Ther. 2014 Sep;31(9):1008-9]. Advances in Therapy, 30(10): 870-84 | no |
|  |  |  | Chandarlapaty S., Chen D., He W., Sung P., Samoila A., You D., Bhatt T., Patel P., Voi M., Gnant M., Hortobagyi G., Baselga J. and Moynahan M. E.. 2016. Prevalence of ESR1 Mutations in Cell-Free DNA and Outcomes in Metastatic Breast Cancer: A Secondary Analysis of the BOLERO-2 Clinical Trial. JAMA oncology, 11: 11 | no |
|  | | | | |
| Bonneterre | excluded | <80% of the study population met the inclusion criteria* | Bonneterre J., Thurlimann B., Robertson J. F., Krzakowski M., Mauriac L., Koralewski P., Vergote I., Webster A., Steinberg M. and von Euler M.. 2000. Anastrozole versus tamoxifen as first-line therapy for advanced breast cancer in 668 postmenopausal women: results of the Tamoxifen or Arimidex Randomized Group Efficacy and Tolerability study.[Erratum appears in J Clin Oncol. 2012 Jan 20;30(3):343]. Journal of Clinical Oncology, 18(22): 3748-57 | no |
|  |  |  | Nabholtz J. M. and Arimidex Study G.. 2003. Advanced breast cancer updates on anastrozole versus tamoxifen. Journal of Steroid Biochemistry & Molecular Biology, 86(3-5): 321-5 | no |
|  |  |  | Nabholtz J. M., Bonneterre J., Buzdar A., Robertson J. F. and Thurlimann B.. 2003. Anastrozole (Arimidex) versus tamoxifen as first-line therapy for advanced breast cancer in postmenopausal women: survival analysis and updated safety results. European journal of cancer (Oxford, England: 2003; 1990) 39(12): 1684-9 | no |
|  |  |  | Nabholtz J. M., Buzdar A., Pollak M., Harwin W., Burton G., Mangalik A., Steinberg M., Webster A. and von Euler M.. 2000. Anastrozole is superior to tamoxifen as first-line therapy for advanced breast cancer in postmenopausal women: results of a North American multicenter randomized trial. Arimidex Study Group. Journal of Clinical Oncology, 18(22): 3758-67 | no |
|  | | | | |
| Buzdar 1996 | excluded | <80% of the study population met the inclusion criteria* | Buzdar A. U., Jonat W., Howell A., Jones S. E., Blomqvist C. P., Vogel C. L., Eiermann W., Wolter J. M., Steinberg M., Webster A. and Lee D.. 1998. Anastrozole versus megestrol acetate in the treatment of postmenopausal women with advanced breast carcinoma: results of a survival update based on a combined analysis of data from two mature phase III trials. Arimidex Study Group.[Erratum appears in Cancer 1999 Feb 15;85(4):1010]. Cancer, 83(6): 1142-52 | no |
|  |  |  | Buzdar A. U., Jones S. E., Vogel C. L., Wolter J., Plourde P. and Webster A.. 1997. A phase III trial comparing anastrozole (1 and 10 milligrams), a potent and selective aromatase inhibitor, with megestrol acetate in postmenopausal women with advanced breast carcinoma. Arimidex Study Group. Cancer, 79(4): 730-9 | no |
|  |  |  | Buzdar A., Jonat W., Howell A., Jones S. E., Blomqvist C., Vogel C. L., Eiermann W., Wolter J. M., Azab M., Webster A. and Plourde P. V.. 1996. Anastrozole, a potent and selective aromatase inhibitor, versus megestrol acetate in postmenopausal women with advanced breast cancer: results of overview analysis of two phase III trials. Arimidex Study Group. Journal of Clinical Oncology, 14(7): 2000-11 | no |
|  | | | | |
| Buzdar 2001 | excluded | <80% of the study population met the inclusion criteria* | Buzdar A., Douma J., Davidson N., Elledge R., Morgan M., Smith R., Porter L., Nabholtz J., Xiang X. and Brady C.. 2001. Phase III, multicenter, double-blind, randomized study of letrozole, an aromatase inhibitor, for advanced breast cancer versus megestrol acetate. Journal of Clinical Oncology, 19(14): 3357-66 | no |
|  | | | | |
| Buzdar 2002 | excluded | TTP not assessed according to RECIST-citeria** | Buzdar A., Hayes D., El-Khoudary A., Yan S., Lonning P., Lichinitser M., Gopal R., Falkson G., Pritchard K., Lipton A., Wolter K., Lee A., Fly K., Chew R., Alderdice M., Burke K., Eisenber P. and Droloxifene 301 Study G.. 2002. Phase III randomized trial of droloxifene and tamoxifen as first-line endocrine treatment of ER/PgR-positive advanced breast cancer. Breast Cancer Research & Treatment, 73(2): 161-75 | no |
|  | | | | |
| Byrne 1997 | excluded | Single publication and only one endpoint reported | Byrne M. J., Gebski V., Forbes J., Tattersall M. H., Simes R. J., Coates A. S., Dewar J., Lunn M., Flower C., Gill P. G. and Stewart J.. 1997. Medroxyprogesterone acetate addition or substitution for tamoxifen in advanced tamoxifen-resistant breast cancer: a phase III randomized trial. Australian-New Zealand Breast Cancer Trials Group. Journal of Clinical Oncology, 15(9): 3141-8 | no |
|  | | | | |
| CALGB 40302 | included | - | Burstein H. J., Cirrincione C. T., Barry W. T., Chew H. K., Tolaney S. M., Lake D. E., Ma C., Blackwell K. L., Winer E. P. and Hudis C. A.. 2014. Endocrine therapy with or without inhibition of epidermal growth factor receptor and human epidermal growth factor receptor 2: a randomized, double-blind, placebo-controlled phase III trial of fulvestrant with or without lapatinib for postmenopausal women with hormone receptor-positive advanced breast cancer-CALGB 40302 (Alliance). Journal of Clinical Oncology, 32(35): 3959-66 | yes |
| CALGB 40503 | included | - | Dickler M. N., Barry W. T., Cirrincione C. T., Ellis M. J., Moynahan M. E., Innocenti F., Hurria A., Rugo H. S., Lake D. E., Hahn O., Schneider B. P., Tripathy D., Carey L. A., Winer E. P. and Hudis C. A.. 2016. Phase III trial evaluating letrozole as first-line endocrine therapy with or without bevacizumab for the treatment of postmenopausal women with hormone receptor-positive advanced-stage breast cancer: CALGB 40503 (Alliance). Journal of Clinical Oncology, 34(22): 2602-2609 | yes |
|  | | | | |
| Castiglione-Gertsch 1993 | excluded | Single publication and only one endpoint reported | Castiglione-Gertsch M., Pampallona S., Varini M., Cavalli F., Brunner K., Senn H. J., Goldhirsch A. and Metzger U.. 1993. Primary endocrine therapy for advanced breast cancer: to start with tamoxifen or with medroxyprogesterone acetate?. Annals of Oncology, 4(9): 735-40 | no |
|  | | | | |
| CONFIRM | included | - | Di Leo A., Jerusalem G., Petruzelka L., Torres R., Bondarenko I. N., Khasanov R., Verhoeven D., Pedrini J. L., Smirnova I., Lichinitser M. R., Pendergrass K., Garnett S., Lindemann J. P., Sapunar F. and Martin M.. 2010. Results of the CONFIRM phase III trial comparing fulvestrant 250 mg with fulvestrant 500 mg in postmenopausal women with estrogen receptor-positive advanced breast cancer. Journal of clinical oncology : official journal of the American Society of Clinical Oncology, 28(30): 4594-600 | yes (PFS) |
|  |  |  | Di Leo A., Jerusalem G., Petruzelka L., Torres R., Bondarenko I. N., Khasanov R., Verhoeven D., Pedrini J. L., Smirnova I., Lichinitser M. R., Pendergrass K., Malorni L., Garnett S., Rukazenkov Y. and Martin M.. 2014. Final overall survival: fulvestrant 500 mg vs 250 mg in the randomized CONFIRM trial. Journal of the National Cancer Institute, 106(1): djt337 | yes (OS) |
|  | | | | |
| Crawford | excluded | <80% of the study population met the inclusion criteria* | Crawford D. J., George W. D., Smith D. C., Stewart M., Paul J. and Leake R. E.. 1994. Cyclic sequential endocrine therapy for advanced breast cancer using a combination of tamoxifen and megestrol acetate. Oncology, 51 Suppl 1: 13-8 | no |
|  | | | | |
| Cristofanilli 2010 | excluded | Single publication and only one endpoint reported | Cristofanilli M., Valero V., Mangalik A., Royce M., Rabinowitz I., Arena F. P., Kroener J. F., Curcio E., Watkins C., Bacus S., Cora E. M., Anderson E. and Magill P. J.. 2010. Phase II, randomized trial to compare anastrozole combined with gefitinib or placebo in postmenopausal women with hormone receptor-positive metastatic breast cancer. Clinical Cancer Research, 16(6): 1904-14 | no |
|  | | | | |
| Deshmane 2007 | excluded | <80% of the study population met the inclusion criteria* | Deshmane V., Krishnamurthy S., Melemed A. S., Peterson P. and Buzdar A. U.. 2007. Phase III double-blind trial of arzoxifene compared with tamoxifen for locally advanced or metastatic breast cancer. Journal of Clinical Oncology, 25(31): 4967-73 | no |
|  | | | | |
| Dombernowsky 1998 | excluded | <80% of the study population met the inclusion criteria* | Dombernowsky P., Smith I., Falkson G., Leonard R., Panasci L., Bellmunt J., Bezwoda W., Gardin G., Gudgeon A., Morgan M., Fornasiero A., Hoffmann W., Michel J., Hatschek T., Tjabbes T., Chaudri H. A., Hornberger U. and Trunet P. F.. 1998. Letrozole, a new oral aromatase inhibitor for advanced breast cancer: double-blind randomized trial showing a dose effect and improved efficacy and tolerability compared with megestrol acetate. Journal of Clinical Oncology, 16(2): 453-61 | no |
|  | | | | |
| EFECT | excluded | Single publication and only one endpoint reported | Chia S., Gradishar W., Mauriac L., Bines J., Amant F., Federico M., Fein L., Romieu G., Buzdar A., Robertson J. F., Brufsky A., Possinger K., Rennie P., Sapunar F., Lowe E. and Piccart M.. 2008. Double-blind, randomized placebo controlled trial of fulvestrant compared with exemestane after prior nonsteroidal aromatase inhibitor therapy in postmenopausal women with hormone receptor-positive, advanced breast cancer: results from EFECT. Journal of clinical oncology : official journal of the American Society of Clinical Oncology, 26(10): 1664-70 | no |
|  | | | | |
| FACT | included | - | Bergh J., Jonsson P. E., Lidbrink E. K., Trudeau M., Eiermann W., Brattstrom D., Lindemann J. P., Wiklund F. and Henriksson R.. 2012. FACT: an open-label randomized phase III study of fulvestrant and anastrozole in combination compared with anastrozole alone as first-line therapy for patients with receptor-positive postmenopausal breast cancer. Journal of clinical oncology : official journal of the American Society of Clinical Oncology, 30(16): 1919-25 | yes |
|  | | | | |
| FERGI | excluded | Single publication and only one endpoint reported | Krop I. E., Mayer I. A., Ganju V., Dickler M., Johnston S., Morales S., Yardley D. A., Melichar B., Forero-Torres A., Lee S. C., de Boer R., Petrakova K., Vallentin S., Perez E. A., Piccart M., Ellis M., Winer E., Gendreau S., Derynck M., Lackner M., Levy G., Qiu J., He J. and Schmid P.. 2016. Pictilisib for oestrogen receptor-positive, aromatase inhibitor-resistant, advanced or metastatic breast cancer (FERGI): a randomised, double-blind, placebo-controlled, phase 2 trial. Lancet Oncology, 17(6): 811-21 | no |
|  | | | | |
| FINDER1 | excluded | Single publication and only one endpoint reported | Ohno S., Rai Y., Iwata H., Yamamoto N., Yoshida M., Iwase H., Masuda N., Nakamura S., Taniguchi H., Kamigaki S. and Noguchi S.. 2010. Three dose regimens of fulvestrant in postmenopausal Japanese women with advanced breast cancer: results from a double-blind, phase II comparative study (FINDER1). Annals of Oncology, 21(12): 2342-7 | no |
|  | | | | |
| FIRST | excluded | <80% of the study population met the inclusion criteria* | Ellis M. J., Llombart-Cussac A., Feltl D., Dewar J. A., Jasiowka M., Hewson N., Rukazenkov Y. and Robertson J. F.. 2015. Fulvestrant 500 mg Versus Anastrozole 1 mg for the First-Line Treatment of Advanced Breast Cancer: Overall Survival Analysis From the Phase II FIRST Study. Journal of clinical oncology : official journal of the American Society of Clinical Oncology, 33(32): 3781-7 | no |
|  |  |  | Robertson J. F., Lindemann J. P., Llombart-Cussac A., Rolski J., Feltl D., Dewar J., Emerson L., Dean A. and Ellis M. J.. 2012. Fulvestrant 500 mg versus anastrozole 1 mg for the first-line treatment of advanced breast cancer: follow-up analysis from the randomized 'FIRST' study. Breast Cancer Research and Treatment, 136(2): 503-11 | no |
|  |  |  | Robertson J. F., Llombart-Cussac A., Rolski J., Feltl D., Dewar J., Macpherson E., Lindemann J. and Ellis M. J.. 2009. Activity of fulvestrant 500 mg versus anastrozole 1 mg as first-line treatment for advanced breast cancer: results from the FIRST study. Journal of clinical oncology : official journal of the American Society of Clinical Oncology, 27(27): 4530-5 | no |
|  | | | | |
| Gershanovich 1997 | excluded | <80% of the study population met the inclusion criteria* | Gershanovich M., Garin A., Baltina D., Kurvet A., Kangas L. and Ellmen J.. 1997. A phase III comparison of two toremifene doses to tamoxifen in postmenopausal women with advanced breast cancer. Eastern European Study Group. Breast Cancer Research & Treatment, 45(3): 251-62 | no |
|  | | | | |
| Gill 1993 | excluded | <80% of the study population met the inclusion criteria* | Gill P. G., Gebski V., Snyder R., Burns I., Levi J., Byrne M. and Coates A.. 1993. Randomized comparison of the effects of tamoxifen, megestrol acetate, or tamoxifen plus megestrol acetate on treatment response and survival in patients with metastatic breast cancer. Annals of Oncology, 4(9): 741-4 | no |
|  | | | | |
| Goss 2007 | excluded | TTP not assessed according to RECIST-citeria** | Goss P., Bondarenko I. N., Manikhas G. N., Pendergrass K. B., Miller Jr W. H., Langecker P. and Blanchett D.. 2007. Phase III, double-blind, controlled trial of atamestane plus toremifene compared with letrozole in postmenopausal women with advanced receptor-positive breast cancer. Journal of Clinical Oncology, 25(31): 4961-4966 | no |
|  | | | | |
| Hayes 1995 | excluded | <80% of the study population met the inclusion criteria* | Hayes D. F., Van Zyl J. A., Hacking A., Goedhals L., Bezwoda W. R., Mailliard J. A., Jones S. E., Vogel C. L., Berris R. F. and Shemano I.. 1995. Randomized comparison of tamoxifen and two separate doses of toremifene in postmenopausal patients with metastatic breast cancer. Journal of Clinical Oncology, 13(10): 2556-66 | no |
|  | | | | |
| Hayes 1995 (North American Trial) +  Gershanovich 1997 (Eastern European Trial) | excluded | <80% of the study population met the inclusion criteria* | Gershanovich M., Hayes D. F., Ellmen J. and Vuorinen J.. 1997. High-dose toremifene vs tamoxifen in postmenopausal advanced breast cancer. Oncology (Williston Park), 11(5 Suppl 4): 29-36 | no |
|  | | | | |
| Howell 2002 | excluded | Single publication and only one endpoint reported | Howell A., Robertson J. F., Quaresma Albano J., Aschermannova A., Mauriac L., Kleeberg U. R., Vergote I., Erikstein B., Webster A. and Morris C.. 2002. Fulvestrant, formerly ICI 182,780, is as effective as anastrozole in postmenopausal women with advanced breast cancer progressing after prior endocrine treatment. Journal of clinical oncology : official journal of the American Society of Clinical Oncology, 20(16): 3396-403 | no |
|  | | | | |
| Howell 2004 | excluded | <80% of the study population met the inclusion criteria* | Howell A., Robertson J. F., Abram P., Lichinitser M. R., Elledge R., Bajetta E., Watanabe T., Morris C., Webster A., Dimery I. and Osborne C. K.. 2004. Comparison of fulvestrant versus tamoxifen for the treatment of advanced breast cancer in postmenopausal women previously untreated with endocrine therapy: a multinational, double-blind, randomized trial. Journal of clinical oncology : official journal of the American Society of Clinical Oncology, 22(9): 1605-13 | no |
|  | | | | |
| Hyams 2013 | excluded | Single publication and only one endpoint reported | Hyams D. M., Chan A., de Oliveira C., Snyder R., Vinholes J., Audeh M. W., Alencar V. M., Lombard J., Mookerjee B., Xu J., Brown K. and Klein P.. 2013. Cediranib in combination with fulvestrant in hormone-sensitive metastatic breast cancer: a randomized Phase II study. Investigational New Drugs, 31(5): 1345-54 | no |
|  | | | | |
| Ibrahim 2011 | excluded | Single publication and only one endpoint reported | Ibrahim N. K., Yariz K. O., Bondarenko I., Manikhas A., Semiglazov V., Alyasova A., Komisarenko V., Shparyk Y., Murray J. L., Jones D., Senderovich S., Chau A., Erlandsson F., Acton G. and Pegram M.. 2011. Randomized phase II trial of letrozole plus anti-MUC1 antibody AS1402 in hormone receptor-positive locally advanced or metastatic breast cancer. Clinical Cancer Research, 17(21): 6822-30 | no |
|  | | | | |
| Ingle 1986 | excluded | <80% of the study population met the inclusion criteria* | Ingle J. N., Green S. J. and Ahmann D. L.. 1986. Randomized trial of tamoxifen alone or combined with aminoglutethimide and hydrocortisone in women with metastatic breast cancer. Journal of Clinical Oncology, 4(6): 958-964 | no |
|  | | | | |
| Ingle 1988 | excluded | <80% of the study population met the inclusion criteria* | Ingle J. N., Twito D. I., Schaid D. J., Cullinan S. A., Krook J. E., Mailliard J. A., Marschke R. F., Long H. J., Gerstner J. G. and Windschitl H. E.. 1988. Randomized clinical trial of tamoxifen alone or combined with fluoxymesterone in postmenopausal women with metastatic breast cancer. Journal of Clinical Oncology, 6(5): 825-31 | no |
|  |  |  | Ingle J. N., Twito D. I., Schaid D. J., Cullinan S. A., Krook J. E., Mailliard J. A., Tschetter L. K., Long H. J., Gerstner J. G. and Windschitl H. E.. 1991. Combination hormonal therapy with tamoxifen plus fluoxymesterone versus tamoxifen alone in postmenopausal women with metastatic breast cancer. An updated analysis. Cancer, 67(4): 886-91 | no |
|  | | | | |
| Ingle 1991 | excluded | <80% of the study population met the inclusion criteria* | Ingle J. N., Mailliard J. A., Schaid D. J., Krook J. E., Gesme D. H., Jr., Windschitl H. E., Pfeifle D. M., Etzell P. S., Gerstner J. G. and Long H. J.. 1991. A double-blind trial of tamoxifen plus prednisolone versus tamoxifen plus placebo in postmenopausal women with metastatic breast cancer. A collaborative trial of the North Central Cancer Treatment Group and Mayo Clinic. Cancer, 68(1): 34-9 | no |
|  | | | | |
| Ingle 1999 | excluded | PFS not assessed according to RECIST-citeria** | Ingle J. N., Suman V. J., Kardinal C. G., Krook J. E., Mailliard J. A., Veeder M. H., Loprinzi C. L., Dalton R. J., Hartmann L. C., Conover C. A. and Pollak M. N.. 1999. A randomized trial of tamoxifen alone or combined with octreotide in the treatment of women with metastatic breast carcinoma. Cancer, 85(6): 1284-92 | no |
|  | | | | |
| Iwata 2013 | included | - | Iwata H., Masuda N., Ohno S., Rai Y., Sato Y., Ohsumi S., Hashigaki S., Nishizawa Y., Hiraoka M., Morimoto T., Sasano H., Saeki T. and Noguchi S.. 2013. A randomized, double-blind, controlled study of exemestane versus anastrozole for the first-line treatment of postmenopausal Japanese women with hormone-receptor-positive advanced breast cancer. Breast Cancer Research & Treatment, 139(2): 441-51 | yes |
|  | | | | |
| Johnston 2004 | excluded | Single publication and only one endpoint reported;  HR is not reported | Johnston S. R., Gumbrell L. A., Evans T. R., Coleman R. E., Smith I. E., Twelves C. J., Soukop M., Rea D. W., Earl H. M., Howell A., Jones A., Canney P., Powles T. J., Haynes B. P., Nutley B., Grimshaw R., Jarman M., Halbert G. W., Brampton M., Haviland J., Dowsett M., Coombes R. C. and Cancer Research U. K. P. I. I. I. C.. 2004. A cancer research (UK) randomized phase II study of idoxifene in patients with locally advanced/metastatic breast cancer resistant to tamoxifen. Cancer Chemotherapy and Pharmacology, 53(4): 341-8 | no |
|  | | | | |
| Johnston 2009 | excluded | OS (HR+CI) not reported for relevant full population | Johnston S., Pippen J., Jr., Pivot X., Lichinitser M., Sadeghi S., Dieras V., Gomez H. L., Romieu G., Manikhas A., Kennedy M. J., Press M. F., Maltzman J., Florance A., O'Rourke L., Oliva C., Stein S. and Pegram M.. 2009. Lapatinib combined with letrozole versus letrozole and placebo as first-line therapy for postmenopausal hormone receptor-positive metastatic breast cancer. Journal of Clinical Oncology, 27(33): 5538-46 | no |
|  | | | | |
| Klijn 2000 | excluded | <80% of the study population met the inclusion criteria* | Klijn J. G., Beex L. V., Mauriac L., van Zijl J. A., Veyret C., Wildiers J., Jassem J., Piccart M., Burghouts J., Becquart D., Seynaeve C., Mignolet F. and Duchateau L.. 2000. Combined treatment with buserelin and tamoxifen in premenopausal metastatic breast cancer: a randomized study. Journal of the National Cancer Institute, 92(11): 903-11 | no |
|  | | | | |
| LEA | included | - | Martin M., Loibl S., von Minckwitz G., Morales S., Martinez N., Guerrero A., Anton A., Aktas B., Schoenegg W., Munoz M., Garcia-Saenz J. A., Gil M., Ramos M., Margeli M., Carrasco E., Liedtke C., Wachsmann G., Mehta K. and De la Haba-Rodriguez J. R.. 2015. Phase III trial evaluating the addition of bevacizumab to endocrine therapy as first-line treatment for advanced breast cancer: the letrozole/fulvestrant and avastin (LEA) study. Journal of Clinical Oncology, 33(9): 1045-52 | yes |
|  | | | | |
| Llombart-Cussac 2012 | included | - | Llombart-Cussac A., Ruiz A., Anton A., Barnadas A., Antolin S., Ales-Martinez J. E., Alvarez I., Andres R., Garcia Saenz J. A., Lao J., Carrasco E., Camara C., Casas I. and Martin M.. 2012. Exemestane versus anastrozole as front-line endocrine therapy in postmenopausal patients with hormone receptor-positive, advanced breast cancer: final results from the Spanish Breast Cancer Group 2001-03 phase 2 randomized trial. Cancer, 118(1): 241-7 | yes |
|  | | | | |
| Mehta 2012 | included | - | Mehta R. S., Barlow W. E., Albain K. S., Vandenberg T. A., Dakhil S. R., Tirumali N. R., Lew D. L., Hayes D. F., Gralow J. R., Livingston R. B. and Hortobagyi G. N.. 2012. Combination anastrozole and fulvestrant in metastatic breast cancer. New England Journal of Medicine, 367(5): 435-44 | yes |
|  | | | | |
| Milla-Santos 2001 | excluded | TTP not assessed according to RECIST-citeria** | Milla-Santos A., Milla L., Rallo L. and Solano V.. 2001. Phase III randomized trial of toremifene vs tamoxifen in hormonodependant advanced breast cancer. Breast Cancer Research & Treatment, 65(2): 119-24 | no |
|  | | | | |
| Milla-Santos 2003 | excluded | Analyses of TTP not in ITT-analyses set, only for patients with clinical benefit | Milla-Santos A., Milla L., Portella J., Rallo L., Pons M., Rodes E., Casanovas J. and Puig-Gali M.. 2003. Anastrozole versus tamoxifen as first-line therapy in postmenopausal patients with hormone-dependent advanced breast cancer: a prospective, randomized, phase III study. American Journal of Clinical Oncology, 26(3): 317-22 | no |
|  | | | | |
| Mouridsen | excluded | HR with CI (or SE) not reported for all endpoints | Lipton A., Ali S. M., Leitzel K., Demers L., Harvey H. A., Chaudri-Ross H. A., Brady C., Wyld P. and Carney W.. 2003. Serum HER-2/neu and response to the aromatase inhibitor letrozole versus tamoxifen. Journal of Clinical Oncology, 21(10): 1967-72 | no |
|  |  |  | Mouridsen H. and Chaudri-Ross H. A.. 2004. Efficacy of first-line letrozole versus tamoxifen as a function of age in postmenopausal women with advanced breast cancer. Oncologist, 9(5): 497-506 | no |
|  |  |  | Mouridsen H. T.. 2007. Letrozole in advanced breast cancer: the PO25 trial.[Erratum appears in Breast Cancer Res Treat. 2008 Nov;112(2):381], [Erratum appears in Breast Cancer Res Treat. 2007;105 Suppl 1:31]. Breast Cancer Research & Treatment, 105 Suppl 1: 19-29 | no |
|  |  |  | Mouridsen H., Gershanovich M., Sun Y., Perez-Carrion R., Boni C., Monnier A., Apffelstaedt J., Smith R., Sleeboom H. P., Jaenicke F., Pluzanska A., Dank M., Becquart D., Bapsy P. P., Salminen E., Snyder R., Chaudri-Ross H., Lang R., Wyld P. and Bhatnagar A.. 2003. Phase III study of letrozole versus tamoxifen as first-line therapy of advanced breast cancer in postmenopausal women: analysis of survival and update of efficacy from the International Letrozole Breast Cancer Group. Journal of Clinical Oncology, 21(11): 2101-9 | no |
|  |  |  | Mouridsen H., Gershanovich M., Sun Y., Perez-Carrion R., Boni C., Monnier A., Apffelstaedt J., Smith R., Sleeboom H. P., Janicke F., Pluzanska A., Dank M., Becquart D., Bapsy P. P., Salminen E., Snyder R., Lassus M., Verbeek J. A., Staffler B., Chaudri-Ross H. A. and Dugan M.. 2001. Superior efficacy of letrozole versus tamoxifen as first-line therapy for postmenopausal women with advanced breast cancer: results of a phase III study of the International Letrozole Breast Cancer Group.[Erratum appears in J Clin Oncol 2001 Jul 1;19(13):3302]. Journal of Clinical Oncology, 19(10): 2596-606 | no |
|  |  |  | Mouridsen H., Sun Y., Gershanovich M., Perez-Carrion R., Becquart D., Chaudri-Ross H. A. and Lang R.. 2004. Superiority of letrozole to tamoxifen in the first-line treatment of advanced breast cancer: evidence from metastatic subgroups and a test of functional ability. Oncologist, 9(5): 489-96 | no |
|  | | | | |
| Muss 1988 | excluded | <80% of the study population met the inclusion criteria* | Muss H. B., Wells H. B., Paschold E. H., Black W. R., Cooper M. R., Capizzi R. L., Christian R., Cruz J. M., Jackson D. V. and Powell B. L.. 1988. Megestrol acetate versus tamoxifen in advanced breast cancer: 5-year analysis--a phase III trial of the Piedmont Oncology Association. Journal of Clinical Oncology, 6(7): 1098-106 | no |
|  | | | | |
| NCT00327769 | excluded | Single publication and only one endpoint reported | Xu B., Jiang Z., Shao Z., Wang J., Feng J., Song S., Chen Z., Gu K., Yu S., Zhang Y., Wang C., Zhang F. and Yang J.. 2011. Fulvestrant 250 mg versus anastrozole for Chinese patients with advanced breast cancer: results of a multicentre, double-blind, randomised phase III trial. Cancer Chemotherapy & Pharmacology, 67(1): 223-30 | no |
|  | | | | |
| Osborne 2002 | excluded | Single publication and only one endpoint reported | Osborne C. K., Pippen J., Jones S. E., Parker L. M., Ellis M., Come S., Gertler S. Z., May J. T., Burton G., Dimery I., Webster A., Morris C., Elledge R. and Buzdar A.. 2002. Double-blind, randomized trial comparing the efficacy and tolerability of fulvestrant versus anastrozole in postmenopausal women with advanced breast cancer progressing on prior endocrine therapy: results of a North American trial. Journal of Clinical Oncology, 20(16): 3386-95 | no |
|  | | | | |
| Osborne 2011 | excluded | Single publication and only one endpoint reported | Osborne C. K., Neven P., Dirix L. Y., Mackey J. R., Robert J., Underhill C., Schiff R., Gutierrez C., Migliaccio I., Anagnostou V. K., Rimm D. L., Magill P. and Sellers M.. 2011. Gefitinib or placebo in combination with tamoxifen in patients with hormone receptor-positive metastatic breast cancer: a randomized phase II study. Clinical Cancer Research, 17(5): 1147-59 | no |
|  | | | | |
| O'Shaughnessy 2016 | excluded | Single publication and only one endpoint reported | O'Shaughnessy J., Campone M., Brain E., Neven P., Hayes D., Bondarenko I., Griffin T. W., Martin J., De Porre P., Kheoh T., Yu M. K., Peng W. and Johnston S.. 2016. Abiraterone acetate, exemestane or the combination in postmenopausal patients with estrogen receptor-positive metastatic breast cancer. Annals of Oncology, 27(1): 106-13 | no |
|  | | | | |
| PALOMA-1 | included |  | Finn R. S., Crown J. P., Ettl J., Schmidt M., Bondarenko I. M., Lang I., Pinter T., Boer K., Patel R., Randolph S., Kim S. T., Huang X., Schnell P., Nadanaciva S., Bartlett C. H. and Slamon D. J.. 2016. Efficacy and safety of palbociclib in combination with letrozole as first-line treatment of ER-positive, HER2-negative, advanced breast cancer: expanded analyses of subgroups from the randomized pivotal trial PALOMA-1/TRIO-18. Breast Cancer Research, 18(1): 67 | no |
|  |  |  | Finn R. S., Crown J. P., Lang I., Boer K., Bondarenko I. M., Kulyk S. O., Ettl J., Patel R., Pinter T., Schmidt M., Shparyk Y., Thummala A. R., Voytko N. L., Fowst C., Huang X., Kim S. T., Randolph S. and Slamon D. J.. 2015. The cyclin-dependent kinase 4/6 inhibitor palbociclib in combination with letrozole versus letrozole alone as first-line treatment of oestrogen receptor-positive, HER2-negative, advanced breast cancer (PALOMA-1/TRIO-18): a randomised phase 2 study. Lancet Oncology, 16(1): 25-35 | yes |
| PALOMA-3 | excluded | Only one endpoint (PFS) reported | Cristofanilli M., Turner N. C., Bondarenko I., Ro J., Im S. A., Masuda N., Colleoni M., DeMichele A., Loi S., Verma S., Iwata H., Harbeck N., Zhang K., Theall K. P., Jiang Y., Bartlett C. H., Koehler M. and Slamon D.. 2016. Fulvestrant plus palbociclib versus fulvestrant plus placebo for treatment of hormone-receptor-positive, HER2-negative metastatic breast cancer that progressed on previous endocrine therapy (PALOMA-3): final analysis of the multicentre, double-blind, phase 3 randomised controlled trial. The Lancet Oncology, 17(4): 425-439 | no |
|  |  |  | Turner N. C., Ro J., Andre F., Loi S., Verma S., Iwata H., Harbeck N., Loibl S., Bartlett C. H., Zhang K., Giorgetti C., Randolph S., Koehler M. and Cristofanilli M.. 2015. Palbociclib in hormone-receptor-positive advanced breast cancer. New England Journal of Medicine, 373(3): 209-219 | no |
|  |  |  | Verma S., Bartlett C. H., Schnell P., DeMichele A. M., Loi S., Ro J., Colleoni M., Iwata H., Harbeck N., Cristofanilli M., Zhang K., Thiele A., Turner N. C. and Rugo H. S.. 2016. Palbociclib in Combination With Fulvestrant in Women With Hormone Receptor-Positive/HER2-Negative Advanced Metastatic Breast Cancer: Detailed Safety Analysis From a Multicenter, Randomized, Placebo-Controlled, Phase III Study (PALOMA-3). Oncologist, 1: 1 | no |
|  | | | | |
| Paridaens 2008 | excluded | PFS not assessed according to RECIST-citeria** | Paridaens R. J., Dirix L. Y., Beex L. V., Nooij M., Cameron D. A., Cufer T., Piccart M. J., Bogaerts J. and Therasse P.. 2008. Phase III study comparing exemestane with tamoxifen as first-line hormonal treatment of metastatic breast cancer in postmenopausal women: the European Organisation for Research and Treatment of Cancer Breast Cancer Cooperative Group. Journal of Clinical Oncology, 26(30): 4883-90 | no |
|  | | | | |
| Peethambaram 1999 | excluded | <80% of the study population met the inclusion criteria* | Peethambaram P. P., Ingle J. N., Suman V. J., Hartmann L. C. and Loprinzi C. L.. 1999. Randomized trial of diethylstilbestrol vs. tamoxifen in postmenopausal women with metastatic breast cancer. An updated analysis. Breast Cancer Research & Treatment, 54(2): 117-22 | no |
|  | | | | |
| Pyrhönen 1997 | excluded | <80% of the study population met the inclusion criteria* | Pyrhonen S., Valavaara R., Modig H., Pawlicki M., Pienkowski T., Gundersen S., Bauer J., Westman G., Lundgren S., Blanco G., Mella O., Nilsson I., Hietanen T., Hindy I., Vuorinen J. and Hajba A.. 1997. Comparison of toremifene and tamoxifen in post-menopausal patients with advanced breast cancer: a randomized double-blind, the 'nordic' phase III study. British Journal of Cancer, 76(2): 270-7 | no |
|  | | | | |
| Robertson 2003 (Howell 2002+ Osborne 2002) | excluded | <80% of the study population met the inclusion criteria* | Howell A., Pippen J., Elledge R. M., Mauriac L., Vergote I., Jones S. E., Come S. E., Osborne C. K. and Robertson J. F.. 2005. Fulvestrant versus anastrozole for the treatment of advanced breast carcinoma: a prospectively planned combined survival analysis of two multicenter trials. Cancer, 104(2): 236-9 | no |
|  |  |  | Robertson J. F. R., Osborne C. K., Howell A., Jones S. E., Mauriac L., Ellis M., Kleeberg U. R., Come S. E., Vergote I., Gertler S., Buzdar A., Webster A. and Morris C.. 2003. Fulvestrant versus anastrozole for the treatment of advanced breast carcinoma in postmenopausal women: a prospective combined analysis of two multicenter trials. Cancer, 98(2): 229-38 | no |
|  | | | | |
| Robertson 2013 | included | - | Robertson J. F. R., Ferrero J. M., Bourgeois H., Kennecke H., de Boer R. H., Jacot W., McGreivy J., Suzuki S., Zhu M., McCaffery I., Loh E., Gansert J. L. and Kaufman P. A.. 2013. Ganitumab with either exemestane or fulvestrant for postmenopausal women with advanced, hormone-receptor-positive breast cancer: A randomised, controlled, double-blind, phase 2 trial. The Lancet Oncology, 14(3): 228-235 | yes |
|  | | | | |
| Rose 2003 | excluded | Single publication and only one endpoint reported | Rose C., Vtoraya O., Pluzanska A., Davidson N., Gershanovich M., Thomas R., Johnson S., Caicedo J. J., Gervasio H., Manikhas G., Ben Ayed F., Burdette-Radoux S., Chaudri-Ross H. A. and Lang R.. 2003. An open randomised trial of second-line endocrine therapy in advanced breast cancer. comparison of the aromatase inhibitors letrozole and anastrozole. European Journal of Cancer, 39(16): 2318-27 | no |
|  | | | | |
| Smith 2003 | excluded | Single publication and only one endpoint reported | Smith I. E.. 2003. Letrozole versus tamoxifen in the treatment of advanced breast cancer and as neoadjuvant therapy. Journal of Steroid Biochemistry & Molecular Biology, 86(3-5): 289-93 | no |
|  | | | | |
| SMS-pa LAR Studie T302 | excluded | CI (or SE) not reported for all relevant endpoints | Sommer H., Prohl-Steimer B., Bajetta E., Haus U., Janni W. and Kay A.. 2001. [A randomized, double-blind, placebo-controlled, phase 3 trial comparing SMS 201-995 pa LAR plus tamoxifen versus tamoxifen plus placebo in women with locally recurrent or metastatic breast cancer]. Zentralblatt fur Gynakologie, 123(10): 557-61 | no |
|  | | | | |
| SoFEA | included | - | Johnston S. R., Kilburn L. S., Ellis P., Dodwell D., Cameron D., Hayward L., Im Y. H., Braybrooke J. P., Brunt A. M., Cheung K. L., Jyothirmayi R., Robinson A., Wardley A. M., Wheatley D., Howell A., Coombes G., Sergenson N., Sin H. J., Folkerd E., Dowsett M., Bliss J. M. and So F. E. A. i.. 2013. Fulvestrant plus anastrozole or placebo versus exemestane alone after progression on non-steroidal aromatase inhibitors in postmenopausal patients with hormone-receptor-positive locally advanced or metastatic breast cancer (SoFEA): a composite, multicentre, phase 3 randomised trial. The Lancet, Oncology. 14(10): 989-98 | yes |
|  | | | | |
| Swiss Group for Clinical Cancer Research SAKK 20/88 | excluded | HR with CI (or SE) not reported for all endpoints | Thurlimann B., Beretta K., Bacchi M., Castiglione-Gertsch M., Goldhirsch A., Jungi W. F., Cavalli F., Senn H. J., Fey M. and Lohnert T.. 1996. First-line fadrozole HCI (CGS 16949A) versus tamoxifen in postmenopausal women with advanced breast cancer. Prospective randomised trial of the Swiss Group for Clinical Cancer Research SAKK 20/88. Annals of Oncology, 7(5): 471-9 | no |
|  | | | | |
| Tredan 2016 | excluded | HR with CI (or SE) not reported for all endpoints | Tredan O., Follana P., Moullet I., Cropet C., Trager-Maury S., Dauba J., Lavau-Denes S., Dieras V., Beal-Ardisson D., Gouttebel M., Orfeuvre H., Stefani L., Jouannaud C., Burki F., Petit T., Guardiola E., Becuwe C., Blot E., Pujade-Lauraine E. and Bachelot T.. 2016. A phase III trial of exemestane plus bevacizumab maintenance therapy in patients with metastatic breast cancer after first-line taxane and bevacizumab: a GINECO group study. Annals of Oncology, 27(6): 1020-9 | no |
|  | | | | |
| Wolff 2013 | excluded | <80% of the study population met the inclusion criteria* | Wolff A. C., Lazar A. A., Bondarenko I., Garin A. M., Brincat S., Chow L., Sun Y., Neskovic-Konstantinovic Z., Guimaraes R. C., Fumoleau P., Chan A., Hachemi S., Strahs A., Cincotta M., Berkenblit A., Krygowski M., Kang L. L., Moore L. and Hayes D. F.. 2013. Randomized phase III placebo-controlled trial of letrozole plus oral temsirolimus as first-line endocrine therapy in postmenopausal women with locally advanced or metastatic breast cancer. Journal of Clinical Oncology, 31(2): 195-202 | no |
|  | | | | |
| Yamamoto 2013 | included | - | Yamamoto Y., Ishikawa T., Hozumi Y., Ikeda M., Iwata H., Yamashita H., Toyama T., Chishima T., Saji S., Yamamoto-Ibusuki M. and Iwase H.. 2013. Randomized controlled trial of toremifene 120 mg compared with exemestane 25 mg after prior treatment with a non-steroidal aromatase inhibitor in postmenopausal women with hormone receptor-positive metastatic breast cancer. BMC Cancer, 13: 239 | yes |
|  | | | | |
| Yardley 2013 | included | - | Yardley D. A., Ismail-Khan R. R., Melichar B., Lichinitser M., Munster P. N., Klein P. M., Cruickshank S., Miller K. D., Lee M. J. and Trepel J. B.. 2013. Randomized phase II, double-blind, placebo-controlled study of exemestane with or without entinostat in postmenopausal women with locally recurrent or metastatic estrogen receptor-positive breast cancer progressing on treatment with a nonsteroidal aromatase inhibitor. Journal of Clinical Oncology, 31(17): 2128-35 | yes |
|  | | | | |
| ZAMBONEY | included | - | Addison C. L., Pond G. R., Cochrane B., Zhao H., Chia S. K., Levine M. N. and Clemons M.. 2015. Correlation of baseline biomarkers with clinical outcomes and response to fulvestrant with vandetanib or placebo in patients with bone predominant metastatic breast cancer: An OCOG ZAMBONEY sub-study. Journal of Bone Oncology, 4(2): 47-53 | no |
|  |  |  | Clemons M. J., Cochrane B., Pond G. R., Califaretti N., Chia S. K., Dent R. A., Song X., Robidoux A., Parpia S., Warr D., Rayson D., Pritchard K. I. and Levine M. N.. 2014. Randomised, phase II, placebo-controlled, trial of fulvestrant plus vandetanib in postmenopausal women with bone only or bone predominant, hormone-receptor-positive metastatic breast cancer (MBC): the OCOG ZAMBONEY study. Breast Cancer Research and Treatment, 146(1): 153-62 | yes |
|  | | | | |
| Zhang 2016 | excluded | Single publication and only one endpoint reported | Zhang Q., Shao Z., Shen K., Li L., Feng J., Tong Z., Gu K., Wang X., Xu B., Sun G., Chen H., Rukazenkov Y. and Jiang Z.. 2016. Fulvestrant 500 mg vs 250 mg in postmenopausal women with estrogen receptor-positive advanced breast cancer: a randomized, double-blind registrational trial in China. Oncotarget, 23: 23 | no |
| * According to IQWiG methodology it is required that relevant studies reflect the defined patient population of interest in at least 80% according to the inclusion criteria.  ** Trials with TTP or comparable endpoints were considered if the definition was identical to PFS (time from randomization to objective disease progression or death from any cause). Only studies reporting PFS according to Response Evaluation Criteria In Solid Tumors (RECIST) were included to ensure standardized and comparable endpoint evaluation. | | | | |
